# Dietary date palm seed nanoparticles modulate biochemical parameters, immune-antioxidant gene expression, and histomorphology in Nile tilapia

**DOI:** 10.1038/s41598-026-43559-9

**Published:** 2026-04-09

**Authors:** Karima A. Bakry, Haitham G. Abo-Al-Ela, Abdullah A. A. Alghamdi, Mohsen A. Khormi, Rehab H. Moneeb, Mariana S. Alfons, Fatma A. Madkour, Asmaa Y. Wahman, Mahmoud M. S. Farrag, Walaa F. A. Emeish

**Affiliations:** 1 Medicine of Aquatic Life Department, Faculty of Veterinary Medicine, Qena University, Qena, 83523 Egypt; 2 Genetics and Biotechnology, Department of Aquaculture, Faculty of Fish Resources, Suez University, Suez, 43221 Egypt; 3 Genetics and Genetic Engineering, Department of Animal Wealth Development, Faculty of Veterinary Medicine, Badr University in Cairo (BUC), Badr City, Cairo 11829 Egypt; 4 Department of Biology, Faculty of Science, Al-Baha University, Al-Baha, Saudi Arabia; 5 Department of Biology, College of Science, Jazan University, P.O. Box. 114, 45142 Jazan, Saudi Arabia; 6 Zoology Department, Faculty of Science, New Valley University, El Kharga, New Valley 72511 Egypt; 7 Department of Anatomy and Embryology, Faculty of Veterinary Medicine, Qena University, Qena, 83523 Egypt; 8 Chemistry Department, Faculty of Science, New Valley University, El Kharga, New Valley 72511 Egypt; 9 Zoology Department, Faculty of Science, Al-Azhar University, Assiut, Egypt

**Keywords:** Antioxidant status, Feed additives, Green nanoparticles, Immune markers, *Oreochromis niloticus*, *Phoenix dactylifera*, Biochemistry, Biotechnology, Immunology, Physiology, Zoology

## Abstract

Nanotechnology offers promising applications in aquaculture by enhancing drug delivery and improving the efficacy of medicinal plant compounds. This study evaluated the effects of dietary supplementation with date palm seed nanoparticles (DPS-NPs) at 0 (control), 40, 80, and 120 mg/kg diet on serum biochemical parameters, gene expression, and histomorphology in Nile tilapia over a 30-day period. The results revealed a clear positive response across biochemical, molecular, and histological markers. Serum analysis revealed significant increases in total protein and globulin at doses of 80 mg/kg (*p* < 0.05) and 120 mg/kg (*p* < 0.0001), while albumin levels were elevated only at 120 mg/kg (*p* < 0.05). Marked reductions in the oxidative stress marker MDA were observed at both 80 and 120 mg/kg. In addition, hepatic enzymes (ALT and AST) and renal function markers (creatinine and uric acid) were significantly decreased at 120 mg/kg (*p* < 0.05 for ALT and AST; *p* < 0.0001 for creatinine; *p* < 0.05 for uric acid), indicating improved liver and kidney function. Gene expression analysis showed consistent upregulation of *il1β*, *tnfα*, and *tgfβ* in the intestine, liver, and spleen, with peak expression in the intestine at 120 mg/kg (*p* < 0.0001). Furthermore, innate immune genes (*β-defensin 1*, *nkl*) and antioxidant genes (*sod2*, *cat*) were significantly upregulated, reaching their highest expression levels at 120 mg/kg (*p* < 0.0001). Histological observations further confirmed enhanced intestinal villus architecture, increased goblet cell activity, proliferative melano-macrophage centers in the spleen, and restored hepatic structure with active Kupffer cells and telocytes. Collectively, these findings demonstrate that dietary supplementation with DPS-NPs, particularly at 120 mg/kg, strengthens immunity, enhances antioxidant defenses, and improves tissue integrity in Nile tilapia, highlighting its potential as a sustainable functional feed additive in aquaculture.

## Introduction

Nile tilapia (*Oreochromis niloticus*) is one of the most important farmed fish species worldwide due to its adaptability, rapid growth, and substantial contribution to national food security^1,2^. However, intensive aquaculture practices often expose fish to environmental stressors and pathogenic infections, leading to suppressed immunity and reduced productivity^3–5^. To address these challenges, growing attention has been directed toward safe, cost-effective, and environmentally friendly immunostimulants, with particular emphasis on naturally derived compounds^6–8^.

The use of plant materials in nano-form has emerged as a promising strategy, as nanoparticles often enhance the bioavailability, stability, and biological activity of bioactive compounds^9^. Herbal medicine has long been recognized as a major source of therapeutic agents, with many modern pharmaceuticals tracing their origins to plant-derived ingredients. These botanical compounds have significantly contributed to the pharmaceutical sector across diverse applications^10^. In recent years, herbal remedies have gained popularity as primary healthcare approaches due to their minimal side effects, a trend that has extended beyond human medicine into veterinary practices worldwide^11–14^.

In aquaculture, medicinal plants such as *Bambusa vulgaris*, *Moringa oleifera*, and *Manihot esculenta*, which are rich in bioactive compounds, have demonstrated considerable potential for enhancing fish immunity. These compounds act as immunostimulants, reinforcing both innate (non-specific) and adaptive (specific) immune responses^15,16^. Since the immune system is the primary biological defense against pathogens, medicinal plants play a crucial role in improving disease resistance and overall fish health^16,17^. Incorporating such plants into aquafeeds can therefore reduce production losses by preventing disease outbreaks before they occur^18^.

Among the various plants of interest, the date palm (*Phoenix dactylifera* L., Family: Arecaceae) is the oldest cultivated fruit tree worldwide. In the Middle East and North Africa, it plays a vital role in both diets and economies, with its fruits serving as a major source of nutrition—particularly carbohydrates and B-complex vitamins—as well as income^19^. Beyond their nutritional value, date fruits are rich in phenolic antioxidants with well-documented health benefits^20–22^. Date palm seeds (DPS), or pits—often considered agricultural waste—make up about 10–15% of the fruit’s weight and are themselves a valuable source of dietary fiber, iron, and antioxidants^23,24^. They also contain functional nutrients, including fats, proteins, ash, vitamins, and high levels of phenolic compounds^25^.

Traditionally, DPS have been used as animal feed or processed into caffeine-free coffee substitutes^26^. In folk medicine, they have been employed to manage diabetes, liver disorders, and gastrointestinal issues^27^. Experimental studies have shown that DPS extracts reduce gastric ulcers in rats^28^, exhibit anti-inflammatory effects in arthritis models^29^, and improve lipid profiles when administered as defatted^30^.

Conventional date palm seed products, such as seed meals and extracts, have been shown to confer multiple health benefits when incorporated into the diets of Nile tilapia and other aquaculture species. These benefits include enhanced growth performance, improved hematological parameters, and strengthened immune function, effects that are largely attributed to their rich phenolic and flavonoid composition^24^. Moreover, dietary supplementation with date palm extracts has demonstrated antibacterial and immunostimulatory activities in Nile tilapia challenged with *Streptococcus agalactiae*^31^. More recently, date palm seed nanoparticles (DPS-NPs) have attracted attention for their potential to enhance biological effects. Nanoparticle formulations can improve solubility, absorption, and cellular uptake, allowing for better interaction with immune cells. They also exhibit increased accumulation in targeted tissues compared to conventional plant extracts^9^.

Nanoparticle supplementation in aquaculture offers substantial opportunities alongside notable challenges. Current research emphasizes improved nutrient bioavailability, enhanced disease resistance, and increased sustainability^32^. However, concerns regarding nanoparticle toxicity, bioaccumulation, and ecological effects in aquatic systems persist, highlighting the need for rigorous risk assessment and regulatory frameworks^32^. Despite these concerns, nanotechnology plays a significant role in advancing global food security through improved productivity and sustainability^33^, with long-term studies in animal models reporting no observable toxicity^34^.

Building on this established bioactivity, the present study focuses on the formulation of date palm seed components into nanoparticles as a novel approach to potentially improve their functional performance, in line with growing evidence that nanoparticle-based delivery systems can enhance the biological effectiveness of natural compounds. Given the limited information available and the need for a comprehensive assessment of DPS-NPs in aquaculture, this study investigates their potential as a natural and eco-friendly feed additive for Nile tilapia. Specifically, it evaluates their effects on immune responses, antioxidant capacity, biochemical parameters, immune-related gene expression, oxidative stress markers, and tissue histopathology. To the best of our knowledge, this is the first study to explore the health-related effects of DPS-NPs in an aquaculture setting.

## Materials and methods

The experimental protocols of this study were conducted in accordance with the relevant guidelines and approved by the Research Ethics Committee of the Faculty of Science, Al-Azhar University, Assiut, Egypt (Approval No. AZHAR 24/2024). The study adhered to the ARRIVE guidelines (https://arriveguidelines.org).

### Date palm seed nanoparticles (DPS-NPs) preparation and characterization

#### Date palm seed powder (DPSP) preparation

Fully mature date palm seeds (Tamr stage) were obtained from a date processing facility in the Kharga region, New Valley Governorate, Egypt. The seeds were air-dried at ambient temperature (25–30 °C) for one week under controlled conditions. After drying, the seeds were finely ground using an electric grinder (Fritsch, Germany) at the Geology Department, Faculty of Science, Assiut University. The ground material was sieved through a 250 μm mesh to obtain a uniform particle size and stored at − 20 °C until further use.

#### Date palm seed nanoparticles (DPS-NPs) synthesis

DPS-NPs were synthesized following a modified protocol adapted from Mostafa et al.^35^. Briefly, 10 g of DPSP was suspended in 100 mL of 19% hydrochloric acid (Sigma-Aldrich, Germany; assay ≥ 99%) in a 250 mL Scott bottle. The mixture was continuously stirred at 1200 rpm on a magnetic hotplate and maintained at 50 °C for five days to induce acid hydrolysis. After hydrolysis, the suspension was centrifuged at 11,600 rpm for 20 min. The resulting supernatant was collected and dried under a laminar flow hood at 50 °C for 24 h to obtain DPS powder. The dried material was repeatedly washed with deionized water until a neutral pH (~ 6) was achieved, followed by re-drying.

#### Date palm seed nanoparticles (DPS-NPs) characterization

The structural and morphological characteristics of DPS-NPs were analyzed using advanced analytical techniques. High-resolution transmission electron microscopy (HR-TEM) was performed with an FEI TECNAI G2 Spirit TWIN microscope (Czech Republic) equipped with a VELETA camera and operated at 80 kV to examine nanoparticle morphology. Surface topography was assessed using scanning electron microscopy (SEM) with a JEOL JSM-5400 LV system (Japan), after gold-coating the samples with a JEOL JTC-1100E ion sputtering unit. Fourier-transform infrared spectroscopy (FTIR) was carried out using a Nicolet 6700 spectrophotometer, with potassium bromide (KBr) pellets scanned over the 4000–400 cm⁻¹ range at a resolution of 4 cm⁻¹ with 16 accumulations, to confirm chemical structure. Particle size distribution and crystallographic properties were evaluated by X-ray diffraction (XRD) using a Philips PW 1710 diffractometer, operated with nickel-filtered Cu Kα radiation (λ = 1.54060 Å) at 40 kV and 40 mA, over a 2θ range of 4–80°.

### Fish collection, acclimation, and husbandry conditions

Apparently healthy Nile tilapia were obtained and acclimated in circular fiberglass tanks connected to a recirculating aquaculture system at the Fish Research Laboratory, Department of Zoology, Faculty of Science, Al-Azhar University, Assiut, Egypt. A total of 144 fish, with a mean body weight of 43.6 ± 2.9 g, were randomly assigned to four dietary treatment groups. Each group was evenly distributed across three flow-through rearing tanks (120 L capacity each), with twelve fish per tank. Continuous aeration was provided using air pumps and air stones.

Prior to the feeding trial, fish were acclimated for 15 days under controlled laboratory conditions. Throughout both the acclimation and experimental periods, Nile tilapia were fed a commercial pelleted diet formulated for tilapia (Skretting, Egypt). The diet was offered twice daily at a feeding rate of 3% of body weight. The basal diet contained 30% crude protein, 6% crude lipid, 5.22% crude fiber, and 9.5% ash, with an estimated nitrogen-free extract of 49.28%, calculated by difference (100 − [crude protein + crude lipid + crude fiber + ash]). The diet provided a gross energy content of 3,900 kcal/kg.

Water quality parameters were monitored daily and maintained within optimal ranges: temperature 28 ± 0.5 °C, pH 7.0, dissolved oxygen 7.5 mg/L, and ammonia < 0.2 mg/L. A 12 h light:12 h dark photoperiod was maintained throughout the study. The experimental design consisted of three replicate tanks per dietary treatment. No clinical signs of disease or mortality were observed during acclimation.

### Dietary treatments

Following acclimation, fish were assigned to dietary groups and subjected to a 30-day feeding trial. Fish were fed twice daily at a rate of 3% of body weight. At the beginning of the trial, the average body weight and length of the fish were 25 ± 1.5 g and 11 ± 0.5 cm, respectively.

**Fig. 1:**
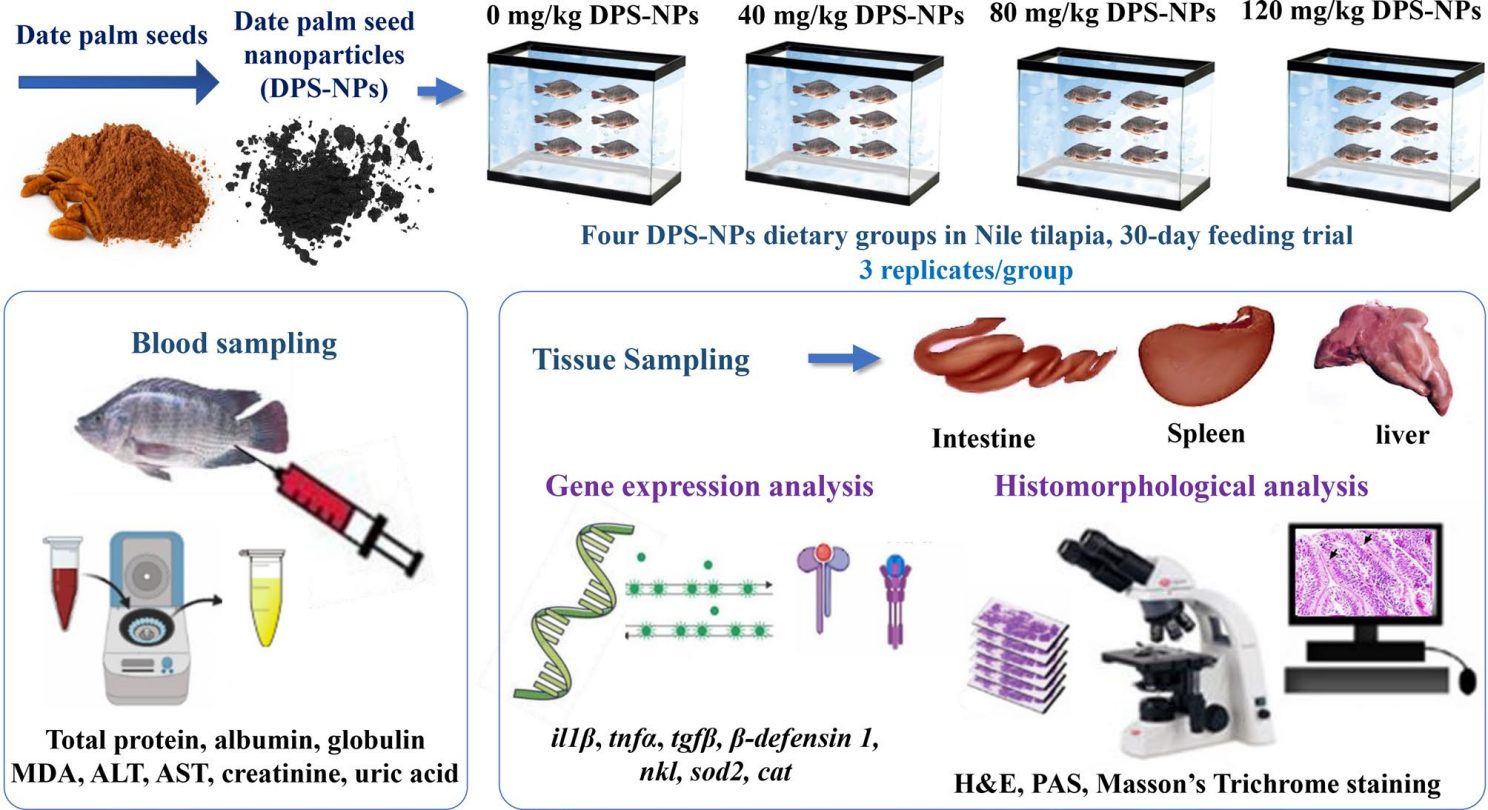
Experimental workflow of dietary treatments with date palm seed nanoparticles (DPS-NPs; 0, 40, 80, and 120 mg/kg) in Nile tilapia over a 30-day feeding trial.

Four diets were formulated: the control group received the basal diet without supplementation, while the three experimental groups were fed the basal diet supplemented with DPS-NPs at 40 mg/kg, 80 mg/kg, and 120 mg/kg diet, respectively. The experimental workflow for the DPS-NPs dietary treatments is illustrated in Fig. 1.

### Blood sampling

At the end of the feeding trial, fish were anesthetized with clove oil at a concentration of 0.05 mL/L^36^. Blood was collected from the caudal vein (*n* = 72) for serum preparation. Samples were left to coagulate at room temperature for 3–4 h, then centrifuged at 4000 rpm for 25 min. The separated serum was carefully harvested, labeled for proper identification, and stored at − 20 °C until further analysis.

### Tissue sampling

Immediately after blood collection, fish were euthanized with an overdose of clove oil (0.5 mL/L). A complete necropsy was performed, and fresh tissue samples from the intestine, spleen, and liver (*n* = 2 per replicate; 6 fish per treatment) were excised. These tissues were immersed in RNA-later solution, refrigerated overnight, and subsequently stored at − 80 °C to preserve RNA integrity for gene expression analysis. Additional tissue samples (*n* = 1 per replicate; 3 fish per treatment) from both the control group and the DPS-NP–supplemented groups were fixed in 10% neutral buffered formalin for histological examination.

### Analysis of serum biochemical indices

For biochemical analysis, serum samples from three fish per replicate were pooled (*n* = 6 pooled samples per group). Biochemical parameters were determined using commercially available kits (Bio-Diagnostic, Worcestershire, UK) according to the manufacturer’s instructions. The following indices were measured: total protein, albumin, creatinine, uric acid, malondialdehyde (MDA), alanine aminotransferase (ALT), and aspartate aminotransferase (AST). All assays were performed using a T80 spectrophotometer (PG Instruments, Leicestershire, UK).

Serum total protein and albumin were measured according to Gornal et al.^37^ and Doumas et al.^38^, respectively, while globulin was calculated as described by Busher^39^. Creatinine and uric acid were analyzed following the methods of Bartels et al.^40^ and Barham and Trinder^41^, respectively. Lipid peroxidation was quantified by determining MDA levels according to Ohkawa et al.^42^. Enzymatic activities of AST and ALT were assessed following Reitman and Frankel^43^.

### Gene expression analysis

#### RNA extraction and cDNA synthesis

Total RNA was isolated from 30 mg of each tissue sample (*n* = 6 per group) using the RNeasy Mini Kit (Qiagen, Hilden, Germany), following the manufacturer’s protocol. RNA concentration and purity were assessed using a NanoDrop LITE Spectrophotometer (Thermo Scientific, USA). Before synthesizing cDNA, RNA integrity was confirmed by evaluating spectrophotometric ratios (A260/A280) and visualizing ribosomal RNA bands via agarose gel electrophoresis. For first-strand complementary DNA (cDNA) synthesis, 1 µg of total RNA was reverse transcribed using the RevertAid First Strand cDNA Synthesis Kit (Thermo Scientific, Waltham, MA, US) according to the manufacturer’s protocol. The resulting cDNA was stored at − 20 °C for subsequent analyses.

#### Quantitative real-time PCR (qRT-PCR)

Tissue-specific primers for Nile tilapia were used in this study, with sequences and details provided in Table 1. The β-actin gene was employed as the internal reference for normalization of expression levels.

**Table 1:** Primers used for real-time PCR.

Gene	Primer name	Primers sequences (5′–3′)	Amplified segment (bp)	Accession number	Reference
Interleukin-1 beta	*il1β* F	TGCACTGTCACTGACAGCCAA	113	DQ061114	^44^
	*il1β* R	ATGTTCAGGTGCACTATGCGG			
Tumor necrosis factor alpha	*tnfα* F	GGAAGCAGCTCCACTCTGATGA	137	JF957373.1	^45^
	*tnfα* R	CACAGCGTGTCTCCTTCGTTCA			
Transforming growth factor beta	*tgfβ* F	GTTTGAACTTCGGCGGTACTG	80	NM_001311325.1	^46^
	*tgfβ* R	TCCTGCTCATAGTCCCAGAGA			
Natural killer-lysin	*nkl* F	ATTTGCGGCACAGTGATTT	162	MF678822	^47^
	*nkl* R	ATGGAAGTCTTGATGGGGCT			
*β*-defensin 1	*β-defensin 1* F	GGTTGTTTTGGCACTTTTGGTT	252	KJ577575	^48^
	*β-defensin 1* R	TGTTGGGAGGCAAACCTTTCT			
Catalase	*cat* F	TCCTGGAGCCTCAGCCAT	79	JF801726	^49^
	*cat* R	ACAGTTATCACACAGGTGCATCTTT			
Superoxide dismutase 2	*sod2* F	CTCCAGCCTGCCCTCAA	58	JF801727.1	^50^
	*sod2* R	TCCAGAAGATGGTGTGGTTAATGTG			
*β*-actin	*β-actin* F	CAGGATGCAGAAGGAGATCACA	92	KJ126772.1	^51^
	*β-actin* R	CGATCCAGACGGAGTATTTACG			

qRT-PCR was performed using the HERAPLUS SYBR Green qPCR Kit (Willowfort, UK) on a CFX96 Real-Time PCR Detection System (Bio-Rad, USA). Primer efficiency was evaluated by standard curve analysis, with all primers showing efficiencies between 90 and 100%. Specificity was confirmed by melt curve analysis (single peaks) and sequencing of PCR products.

The thermal cycling conditions were as follows: for *il1β*, *tgfβ*, *β-defensin 1*, *cat*, *sod2*, and *β-actin*, the program consisted of an initial denaturation at 95 °C for 3 min, followed by 40 cycles of 95 °C for 10 s and annealing/extension at 60 °C for 1 min. For *tnfα* and *nkl*, the program included an initial denaturation at 95 °C for 3 min, followed by 40 cycles of 95 °C for 30 s, 58 °C for 30 s, and 72 °C for 30 s. Fluorescence signals were collected during the extension phase. No-template controls were included in all runs to confirm specificity and exclude contamination.

A post-amplification melt curve analysis was performed from 65 °C to 95 °C, with 0.5 °C increments every 0.05 s, to verify amplification specificity. Cycle threshold (Ct) values were obtained for each reaction, and relative gene expression levels were calculated using the 2^−ΔΔCt^ method^52^.

### Histological procedures and morphometric analysis

Based on the biochemical and gene expression results, the group fed 120 mg/kg DPS-NPs was selected for histological examination. Following fixation in 10% neutral buffered formalin, tissues were dehydrated through a graded ethanol series: 70% (overnight), 80% (2 h), 90% (1 h), and 100% (1 h). Dehydrated samples were cleared in methyl benzoate for two days with two solution changes until translucency was achieved, then embedded in paraffin wax at 65 °C through three infiltration steps (Phase I: 2 h; Phase II: 2 h; Phase III: overnight). After infiltration, samples were oriented and solidified into paraffin blocks.

Paraffin blocks were trimmed and sectioned at a thickness of 3.5–4 μm using a Leica rotary microtome (Leica Microsystems, Wetzlar, Germany). The sections were floated on a 40–45 °C water bath and then mounted onto glass slides. Histological staining was performed using standard hematoxylin and eosin (H&E), as well as Periodic Acid–Schiff (PAS) and Masson’s trichrome stains, following the protocol of Suvarna et al.^53^. Stained sections were cleared in xylene and mounted with Canada balsam. Microscopic examination was conducted using a Leitz Dialux 20 microscope, and photomicrographs were captured with a Canon PowerShot A95 digital camera at 100× and 400× magnifications. Morphometric parameters, including villus height and thickness of the intestinal muscular layer, were quantified using ImageJ software (version 1.54p; Fiji; https://fiji.sc/ and http://imagej.org) and expressed as mean ± SD.

### Statistical analysis

All statistical analyses and graphical outputs were performed using GraphPad Prism (version 9.3.0; San Diego, CA, USA; https://www.graphpad.com/). Data were expressed as mean ± standard error of the mean (SEM). Differences among groups were analyzed by one-way analysis of variance (ANOVA), and significance was considered at *p* < 0.05. Post hoc comparisons were conducted using Tukey’s multiple-range test to identify pairwise differences between treatments. A heatmap with a dendrogram of fold-change data was generated using the expression function within the Heatmapper package (http://www.heatmapper.ca/expression/), applying average linkage hierarchical clustering with the Pearson correlation coefficient distance matrix (default settings). Correlation matrices of gene expression levels in the intestine, spleen, and liver were computed using PAST software (version 4.03; https://past.en.lo4d.com/windows).

## Results

### Characterization of DPS-NPs

**Fig. 2:**
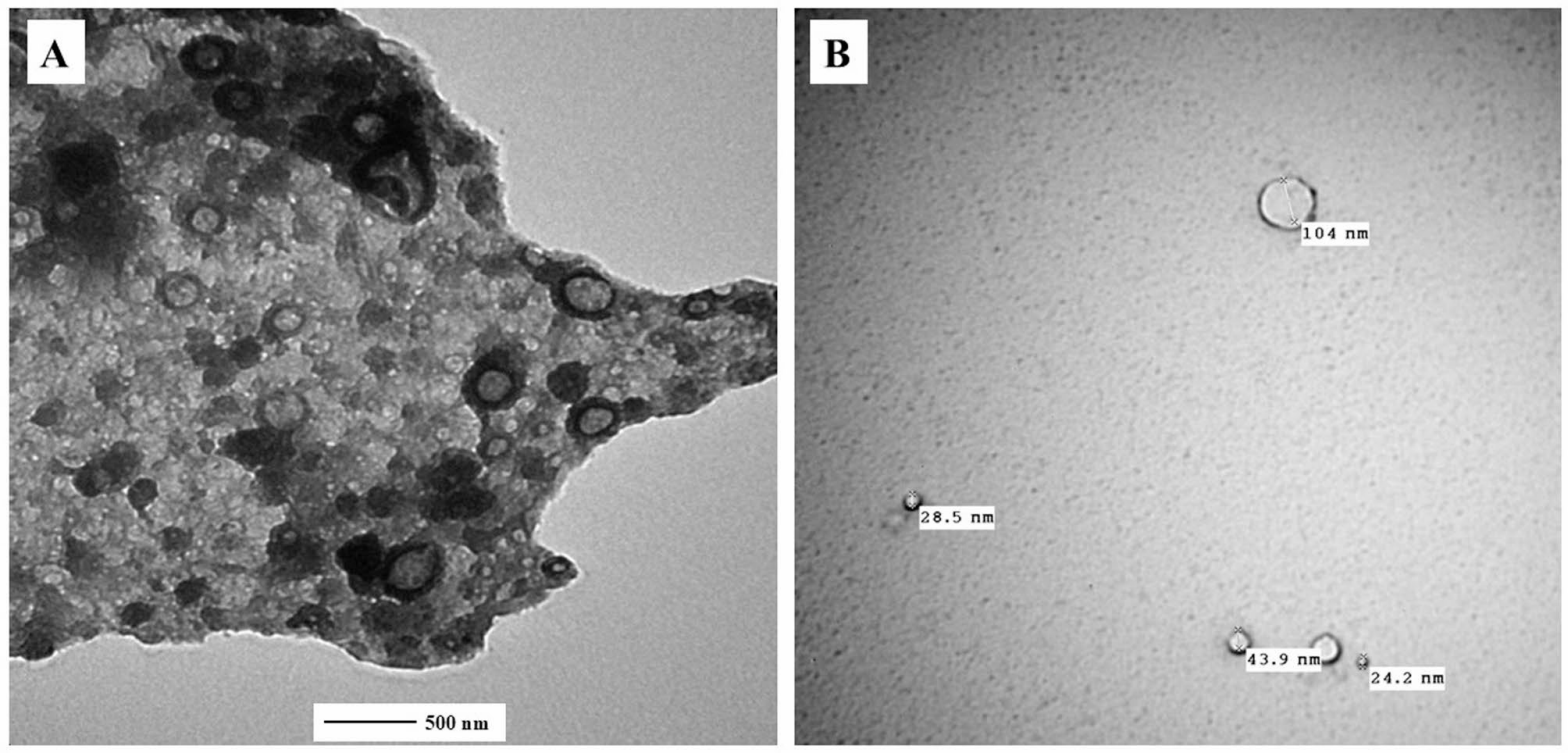
Transmission electron microscopy images of DPS-NPs: **A** agglomerated clusters showing particle aggregation at high magnification (58,000×); **B** dispersed nanoparticles ranging from 24.2–104 nm, with spherical to irregular morphologies.

**Fig. 3:**
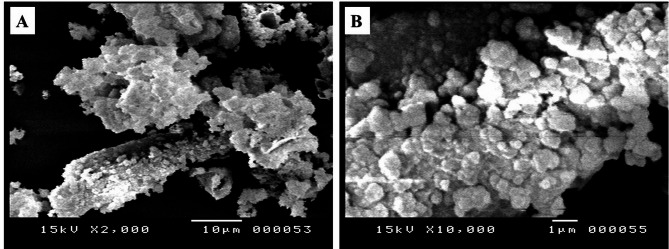
Scanning electron microscopy images of DPS-NPs at two magnifications: **A** low magnification illustrating overall particle distribution and aggregation; **B** high magnification highlighting surface texture and morphological details.

Transmission electron microscopy (TEM) and scanning electron microscopy (SEM) confirmed the successful synthesis of DPS-NPs, which exhibited morphologies ranging from spherical to irregular shapes with particle sizes between 24.2 and 104 nm, along with some degree of aggregation. TEM images (Fig. 2A, B) revealed well-dispersed nanoparticles, while SEM images (Fig. 3A, B) showed a relatively uniform surface distribution and texture. These findings indicate that the applied synthesis method effectively produced DPS-NPs with a high surface area, supporting the conversion of DPS waste into value-added nanoparticles with enhanced bioactive potential.

**Fig. 4:**
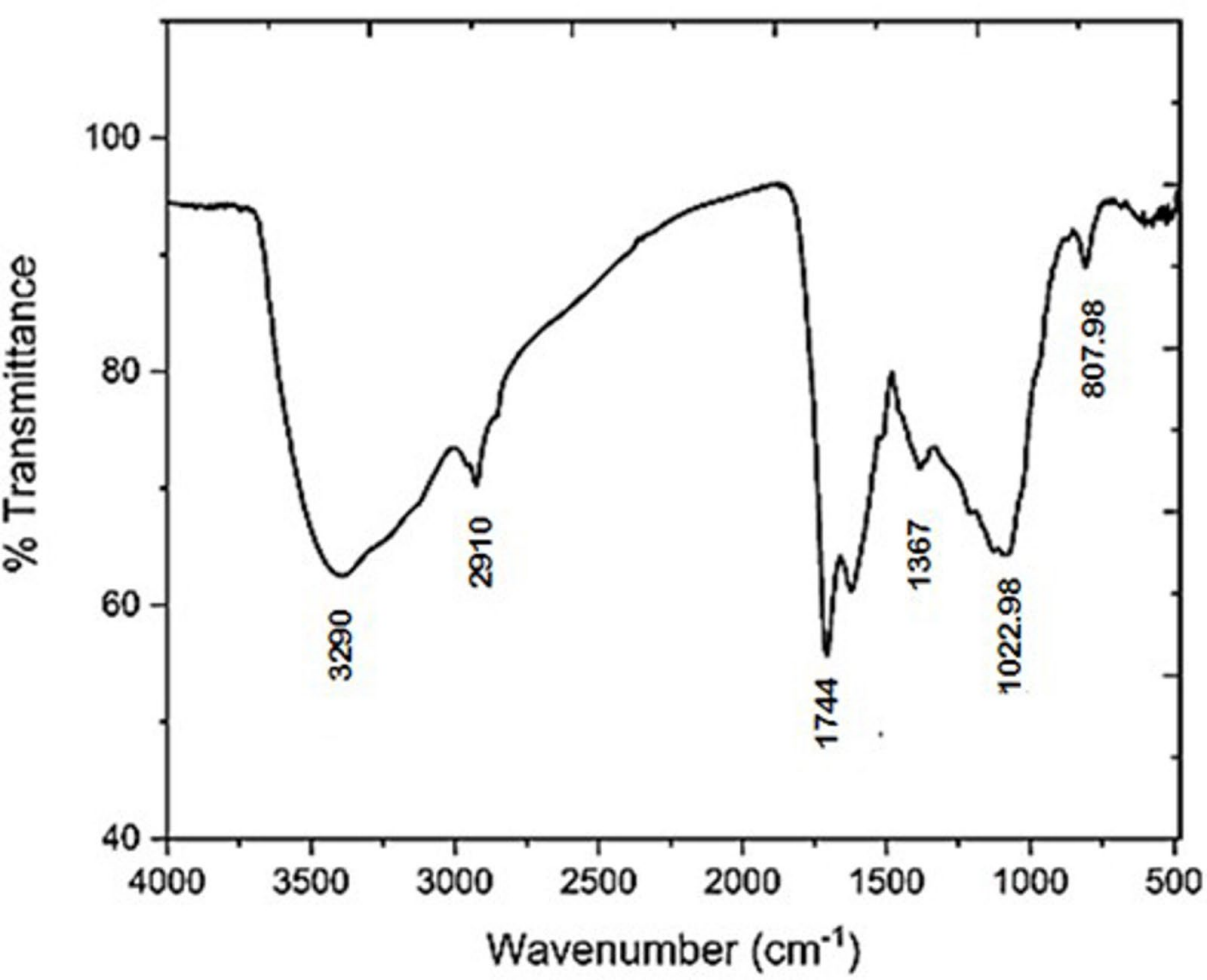
Fourier-transform infrared spectra of synthesized DPS-NPs, showing characteristic absorption bands corresponding to key functional groups.

Fourier-transform infrared (FTIR) spectroscopy (Fig. 4) further confirmed the presence of multiple functional groups. A broad peak at 3290 cm⁻¹ was attributed to hydrogen bonding, likely from hydrates or amino/ammonium groups^35,54,55^. The band at 1367 cm⁻¹ corresponded to hydroxyl group vibrations^54^, while the signal at 2910 cm⁻¹ was assigned to methylene C–H stretching^56,57^. A strong peak at 1744 cm⁻¹ indicated the presence of carbonyl compounds such as esters and ketones^57^. Additional absorption bands included 807.98 cm⁻¹ (aromatic C–H bending) and 1022.98 cm⁻¹ (C–O–C stretching vibrations), the latter typically associated with polysaccharide structures^35,54,57^.

**Fig. 5:**
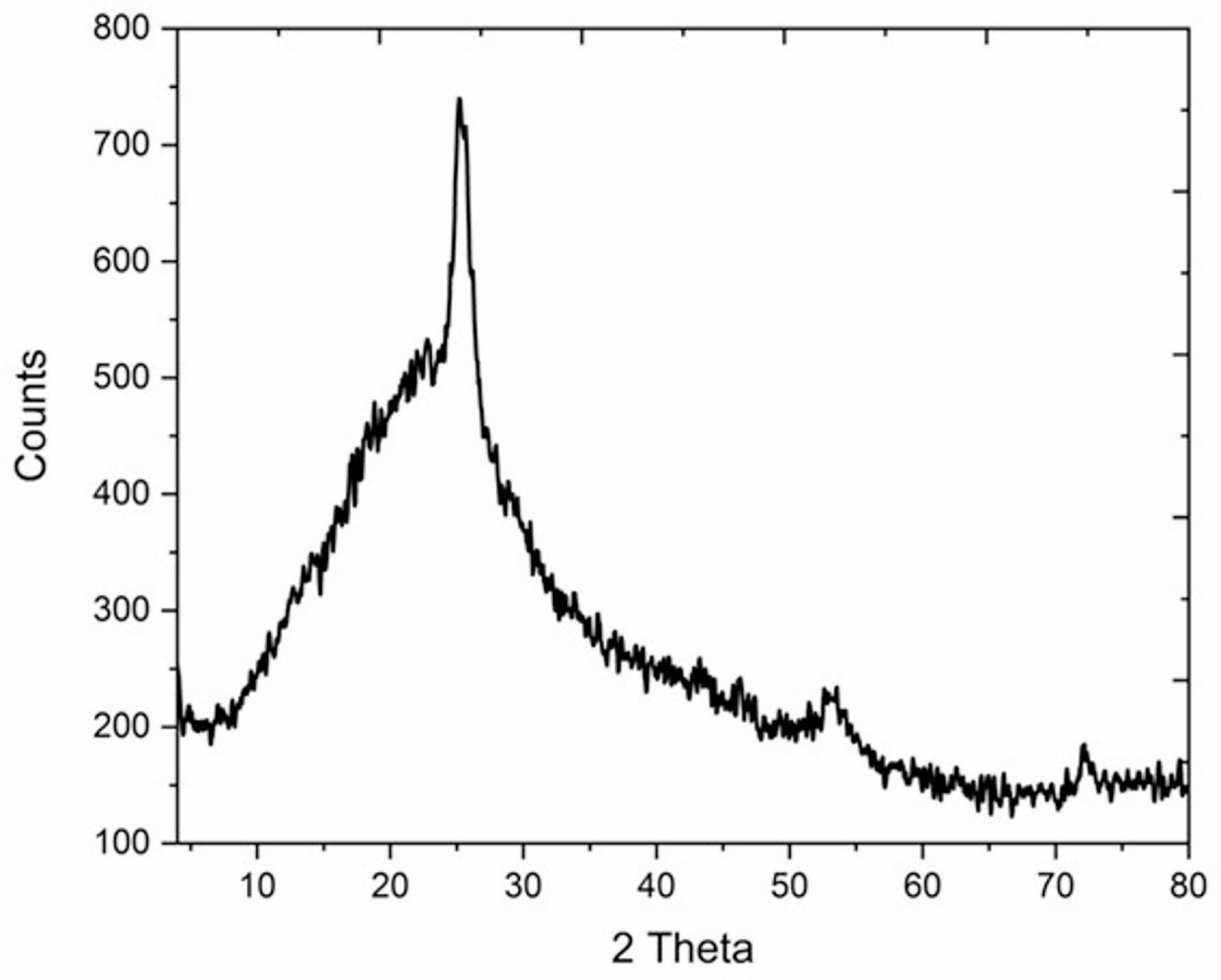
X-ray diffraction pattern of synthesized DPS-NPs, displaying a broad hump centered around 2θ = 25°.

X-ray diffraction (XRD) analysis (Fig. 5) revealed a broad hump centered around 2θ = 25°, with no distinct sharp peaks observed across the 5–80° range, indicating a predominantly amorphous rather than crystalline structure. The broad feature is generally associated with the (002) plane of carbonaceous materials, suggesting the presence of disordered carbon phases derived from organic degradation during nanoparticle synthesis. Such amorphous structures are known to enhance surface reactivity and increase active site availability, thereby improving their potential for applications in adsorption, catalysis, and biomedical fields.

### Serum biochemical profiles

Feeding Nile tilapia with dietary DPS-NPs at 40, 80, and 120 mg/kg for one month produced significant effects on serum biochemical parameters, with significant differences compared to the control group.

**Fig. 6:**
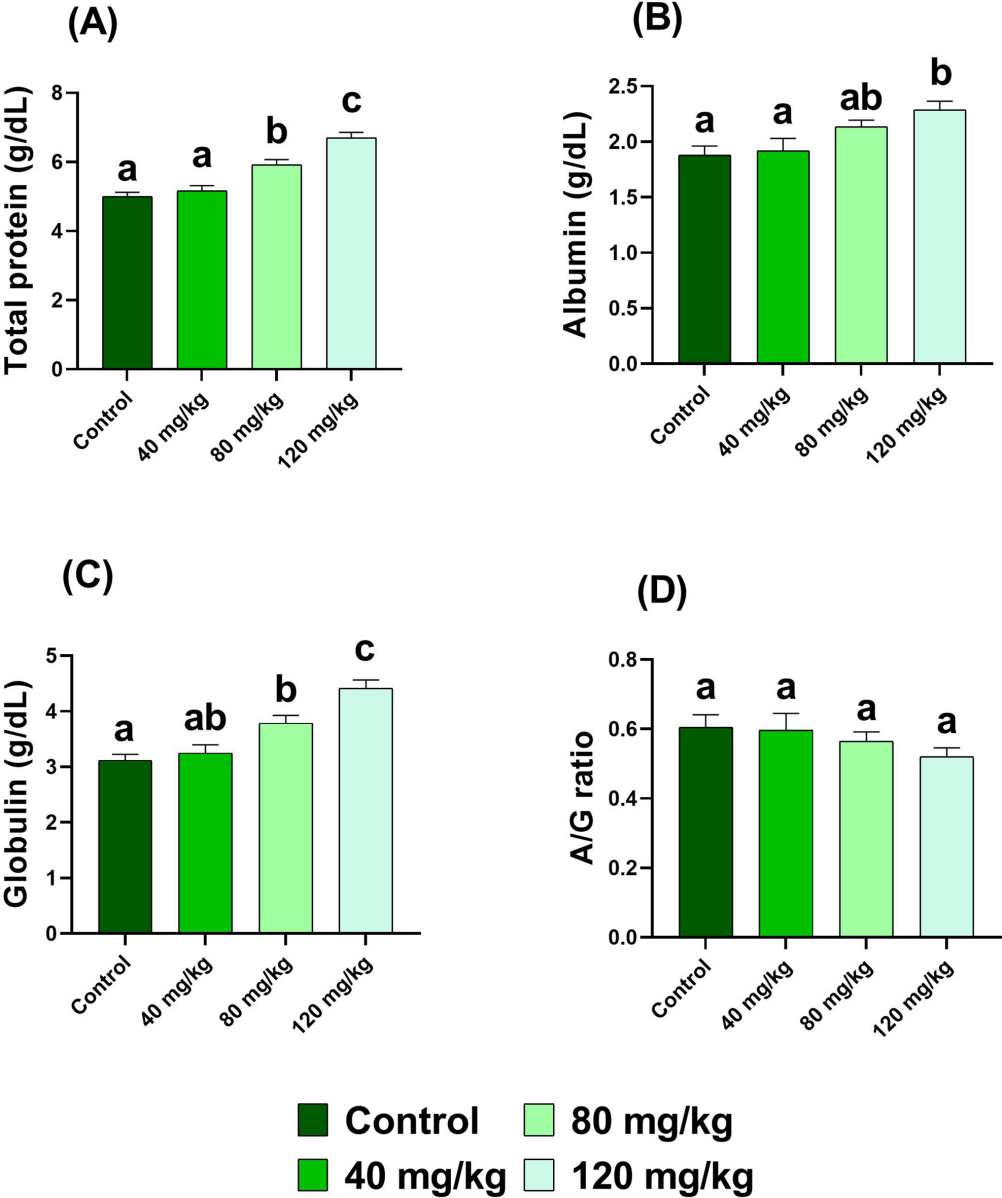
Serum levels of total protein, albumin, globulin, and albumin-to-globulin (A/G) ratio in Nile tilapia fed diets supplemented with DPS-NPs (0, 40, 80, and 120 mg/kg) for one month. Values are expressed as mean ± standard error. Distinct lowercase letters denote statistically significant differences among groups (one-way ANOVA, *p* < 0.05).

Total protein and globulin concentrations increased progressively with supplementation, with significant elevations observed at 80 and 120 mg/kg and the highest values recorded at 120 mg/kg (total protein: 6.7 ± 0.15 g/dL, *p* < 0.0001; globulin: 4.41 ± 0.15 g/dL, *p* < 0.0001). In contrast, albumin levels were significantly elevated only in the 120 mg/kg group (2.3 ± 0.08 g/dL, *p* < 0.05). Despite these changes, the albumin-to-globulin (A/G) ratio remained statistically unchanged across all treatment groups (*p* > 0.05) (Fig. 6).

**Fig. 7:**
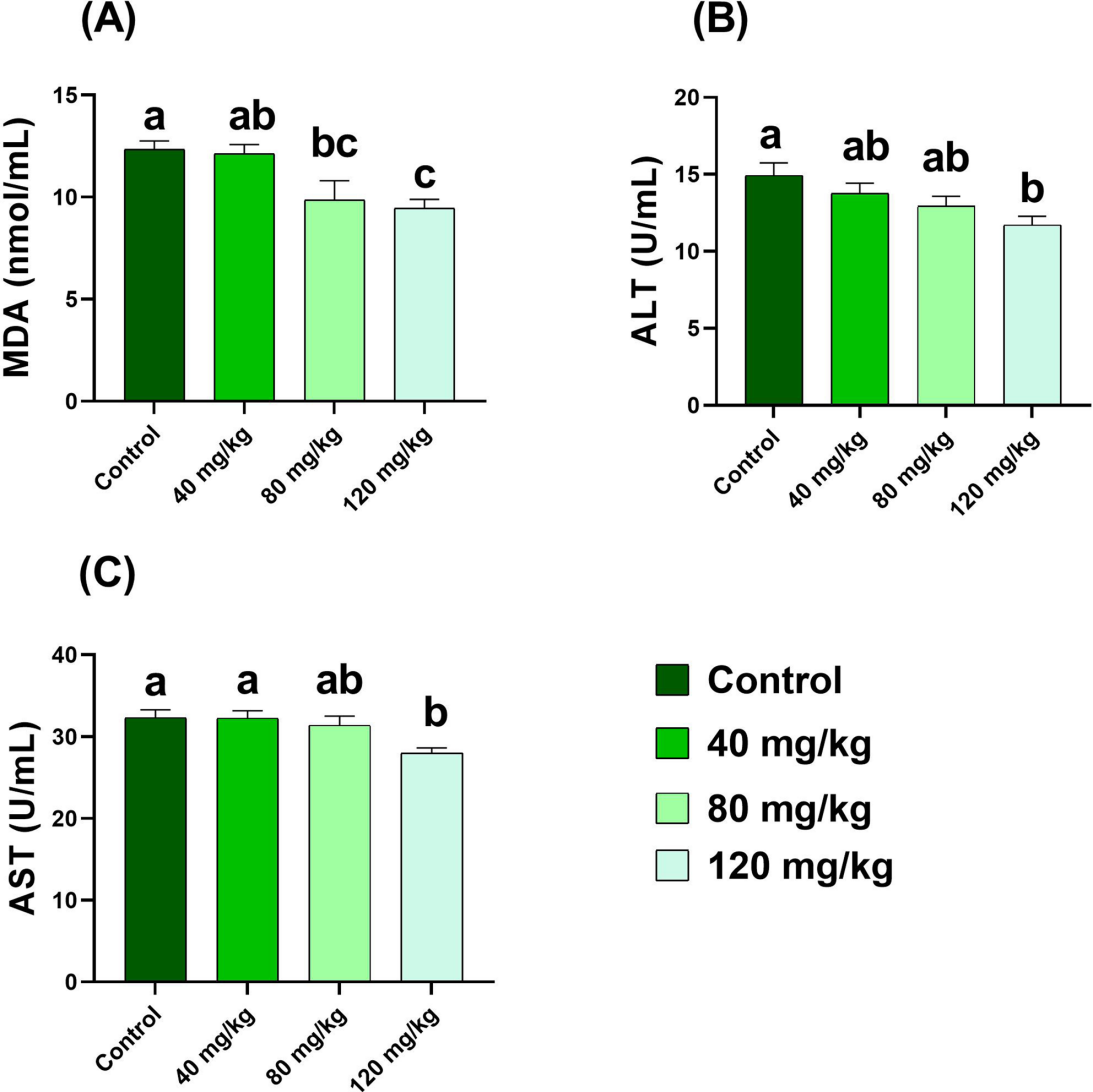
Serum levels of malondialdehyde (MDA), alanine aminotransferase (ALT), and aspartate aminotransferase (AST) in Nile tilapia fed diets supplemented with DPS-NPs (0, 40, 80, and 120 mg/kg) for one month. Values are expressed as mean ± standard error. Distinct lowercase letters denote statistically significant differences among groups (one-way ANOVA, *p* < 0.05).

Serum malondialdehyde (MDA) levels decreased significantly (*p* < 0.05) in the 80 and 120 mg/kg groups, with the lowest concentration recorded at 120 mg/kg (9.45 ± 0.44 nmol/mL). Similarly, the activities of alanine aminotransferase (ALT) and aspartate aminotransferase (AST) were significantly reduced in the 120 mg/kg group (11.7 ± 0.57 U/mL and 28.0 ± 0.62 U/mL, respectively; *p* < 0.05) (Fig. 7).

**Fig. 8:**
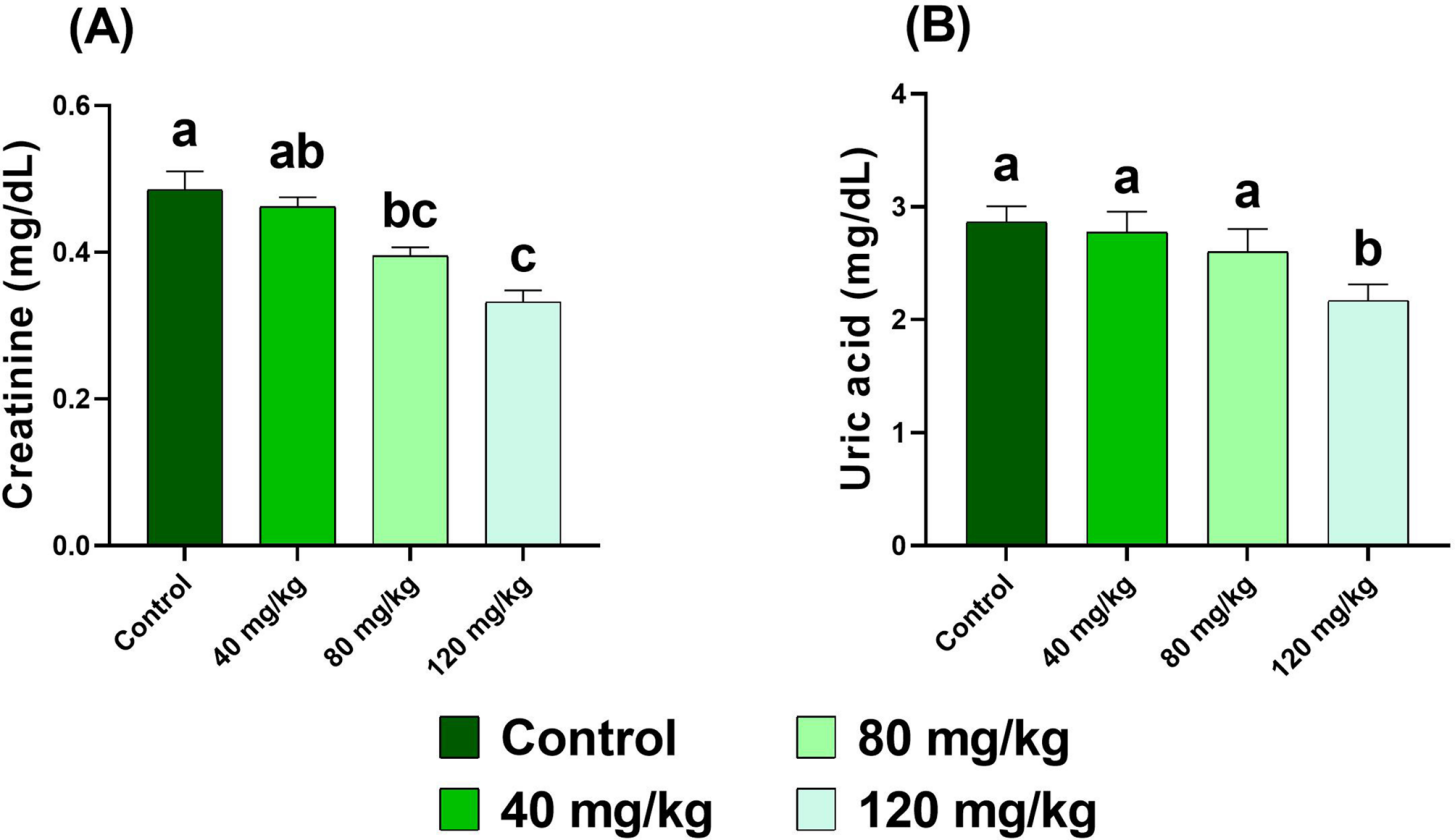
Serum creatinine and uric acid levels in Nile tilapia fed diets supplemented with DPS-NPs (0, 40, 80, and 120 mg/kg) for one month. Values are expressed as mean ± standard error. Distinct lowercase letters denote statistically significant differences among groups (one-way ANOVA, *p* < 0.05).

Creatinine levels declined significantly (*p* < 0.05) in all groups except at 40 mg/kg, with the lowest level observed at 120 mg/kg (0.33 ± 0.01 mg/dL; *p* < 0.0001). Uric acid levels showed a significant reduction only at 120 mg/kg (2.16 ± 0.14 mg/dL, *p* < 0.05), while no significant changes were detected at lower supplementation levels (Fig. 8).

### Gene expression profiles

**Fig. 9:**
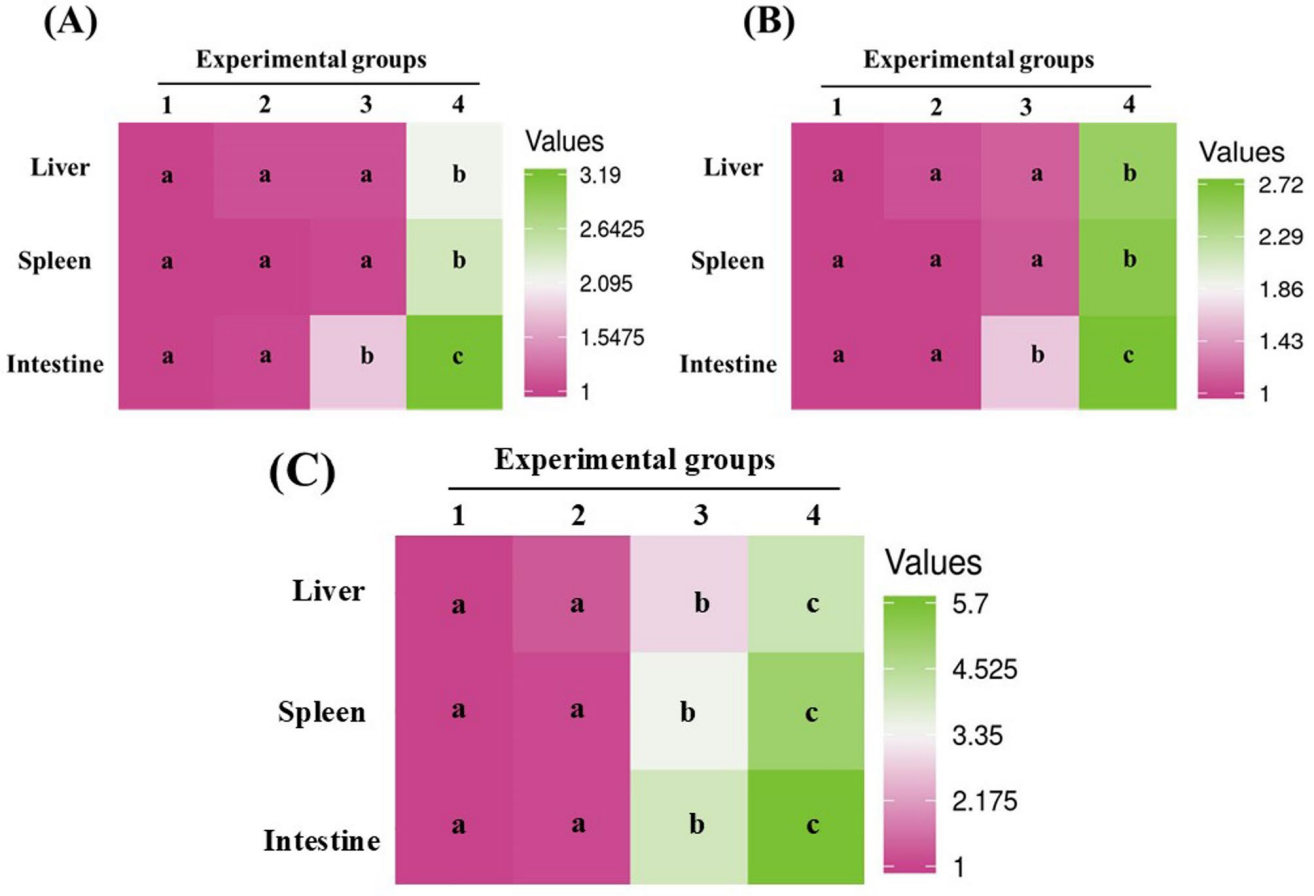
Heatmaps of expression levels of inflammatory cytokines: **A** interleukin-1β (*il1β*), **B** tumor necrosis factor-α (*tnfα*), and **C** transforming growth factor-β (*tgfβ*) in the intestine, spleen, and liver of Nile tilapia fed diets supplemented with DPS-NPs (0, 40, 80, and 120 mg/kg; groups 1–4, respectively) for one month. Distinct lowercase letters denote statistically significant differences among groups per tissue (one-way ANOVA, *p* < 0.05).

In intestinal tissue, supplementation with 80 and 120 mg/kg DPS-NPs significantly upregulated *il1β* and *tnfα*, with the highest fold increases (3.19 and 2.72, respectively; *p* < 0.0001) observed at 120 mg/kg. No significant changes were detected at 40 mg/kg (Fig. 9A, B). In the liver and spleen, significant upregulation of both cytokines was observed exclusively at 120 mg/kg, reaching 2.18- and 2.50-fold in the liver and 2.42- and 2.59-fold in the spleen, respectively (*p* < 0.0001). In contrast, *tgfβ* expression was significantly increased across all examined tissues (intestine, liver, and spleen) at both 80 and 120 mg/kg, with the highest fold changes detected at 120 mg/kg (5.70-, 4.15-, and 4.99-fold, respectively; *p* < 0.0001) (Fig. 9C).

**Fig. 10:**
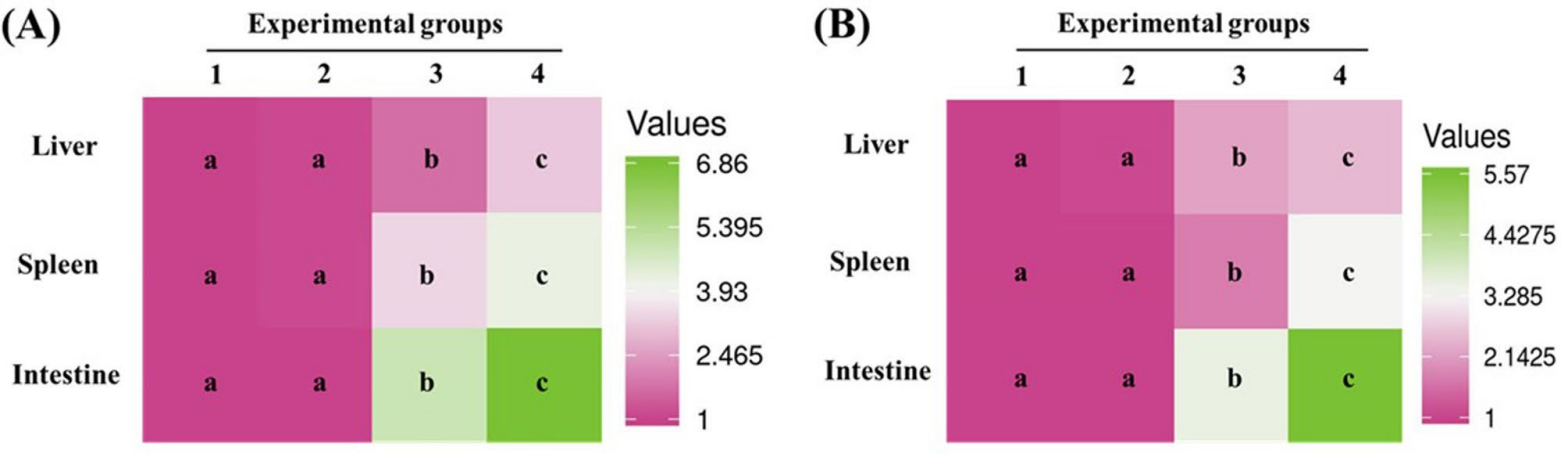
Heatmaps of expression levels of antimicrobial peptides: **A** *β-defensin 1* and **B** NK-lysin (*nkl*) in the intestine, spleen, and liver of Nile tilapia fed diets supplemented with DPS-NPs (0, 40, 80, and 120 mg/kg; groups 1–4, respectively) for one month. Distinct lowercase letters denote statistically significant differences among groups per tissue (one-way ANOVA, *p* < 0.05).

Both *β-defensin 1* and *nkl* were significantly upregulated across all examined tissues at doses of 80 and 120 mg/kg, with maximal induction observed at 120 mg/kg. Specifically, *β-defensin 1* expression increased by 5.57-, 2.50-, and 3.30-fold in the intestine, liver, and spleen, respectively (*p* < 0.0001), while *nkl* expression rose by 6.86-, 3.15-, and 4.19-fold in the same tissues (*p* < 0.0001) (Fig. 10A, B).

**Fig. 11:**
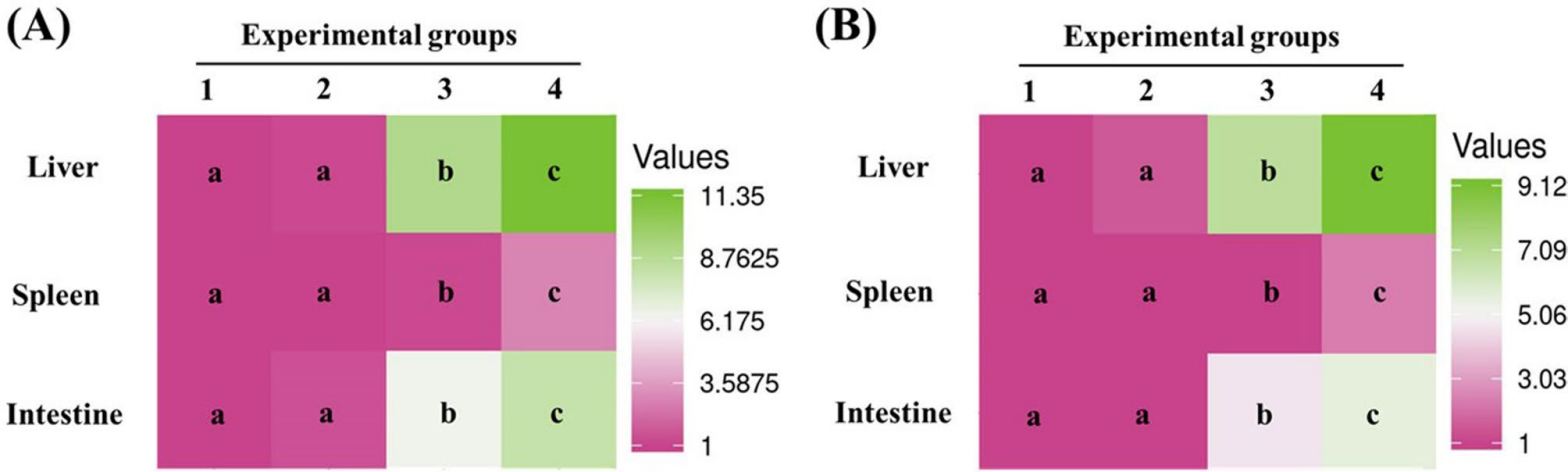
Heatmaps of expression levels of antioxidant enzymes: **A** superoxide dismutase (*sod2*) and **B** catalase (*cat*) in the intestine, spleen, and liver of Nile tilapia fed diets supplemented with DPS-NPs (0, 40, 80, and 120 mg/kg; groups 1–4, respectively) for one month. Distinct lowercase letters denote statistically significant differences among groups per tissue (one-way ANOVA, *p* < 0.05).

Expression of the antioxidant genes *sod2* and *cat* was significantly upregulated in the intestine and liver at doses of 80 and 120 mg/kg, with the highest fold increases observed at 120 mg/kg (*sod2*: 5.64- and 9.12-fold; *cat*: 8.16- and 11.35-fold in the intestine and liver, respectively; *p* < 0.0001). In the spleen, both genes were also significantly upregulated at 80 and 120 mg/kg, with *sod2* and *cat* expression increasing by 2.34- and 3.04-fold, respectively (*p* < 0.0001) (Fig. 11A, B).

**Fig. 12:**
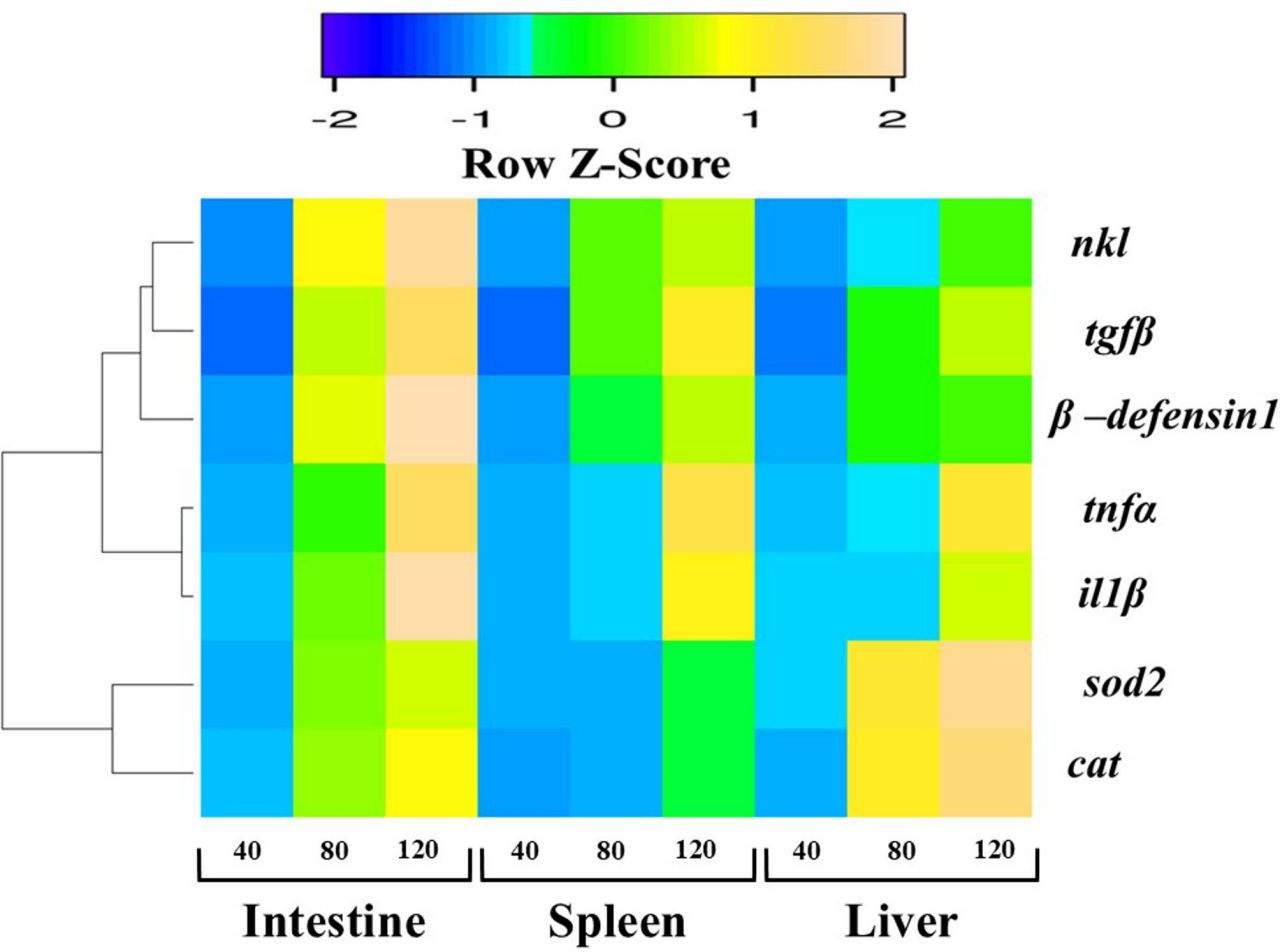
Heatmap with row hierarchical clustering of *il1β*, *tnfα*, *tgfβ*, *nkl*, *β-defensin 1*, *cat*, and *sod2* genes in the intestine, spleen, and liver of Nile tilapia fed DPS-NPs (0, 40, 80, and 120 mg/kg) for one month. Expression values are represented as a color scale from blue (low) to yellow (high).

**Fig. 13:**
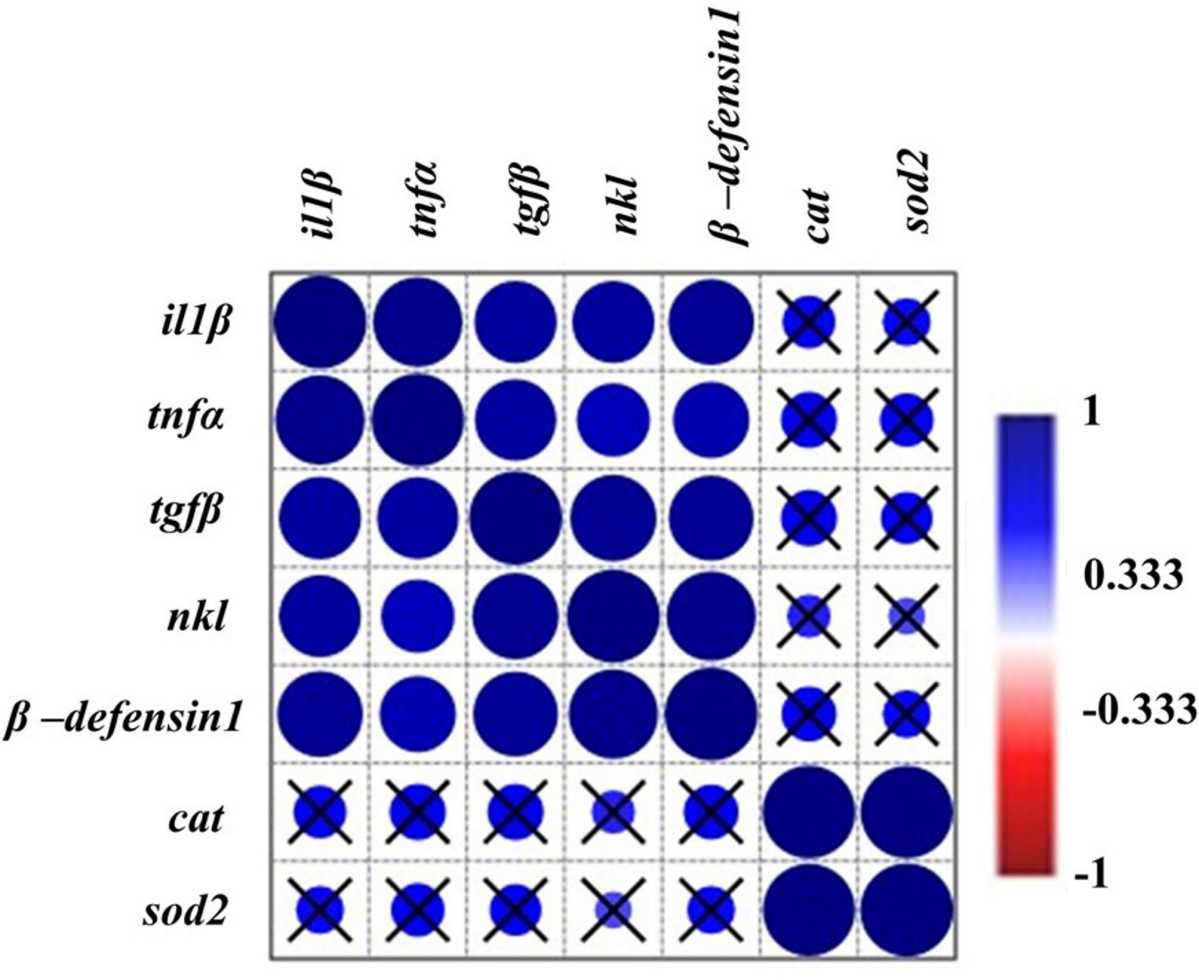
Correlation matrix of *il1β*, *tnfα*, *tgfβ*, *nkl*, *β-defensin 1*, *cat*, and *sod2* gene expression in the intestine, spleen, and liver of Nile tilapia fed DPS-NPs (0, 40, 80, and 120 mg/kg) for one month. Circle size and color intensity represent the strength of correlation (blue = positive, red = negative). Crossed (×) circles indicate non-significant correlations (*P* > 0.05).

Hierarchical cluster analysis (Fig. 12) revealed two main clusters: one containing antioxidant markers (*sod2* and *cat*) and another comprising immune-related genes. Within the immune gene cluster, *il1β* and *tnfα* clustered together, whereas *tgfβ*, *nkl*, and *β-defensin 1* formed a distinct subgroup. Furthermore, correlation analysis (Fig. 13) revealed that all examined genes were positively correlated. Notably, *sod2* and *cat* exhibited a statistically significant positive correlation with each other, whereas their correlations with the other studied genes, though positive, were not statistically significant.

**Fig. 14:**
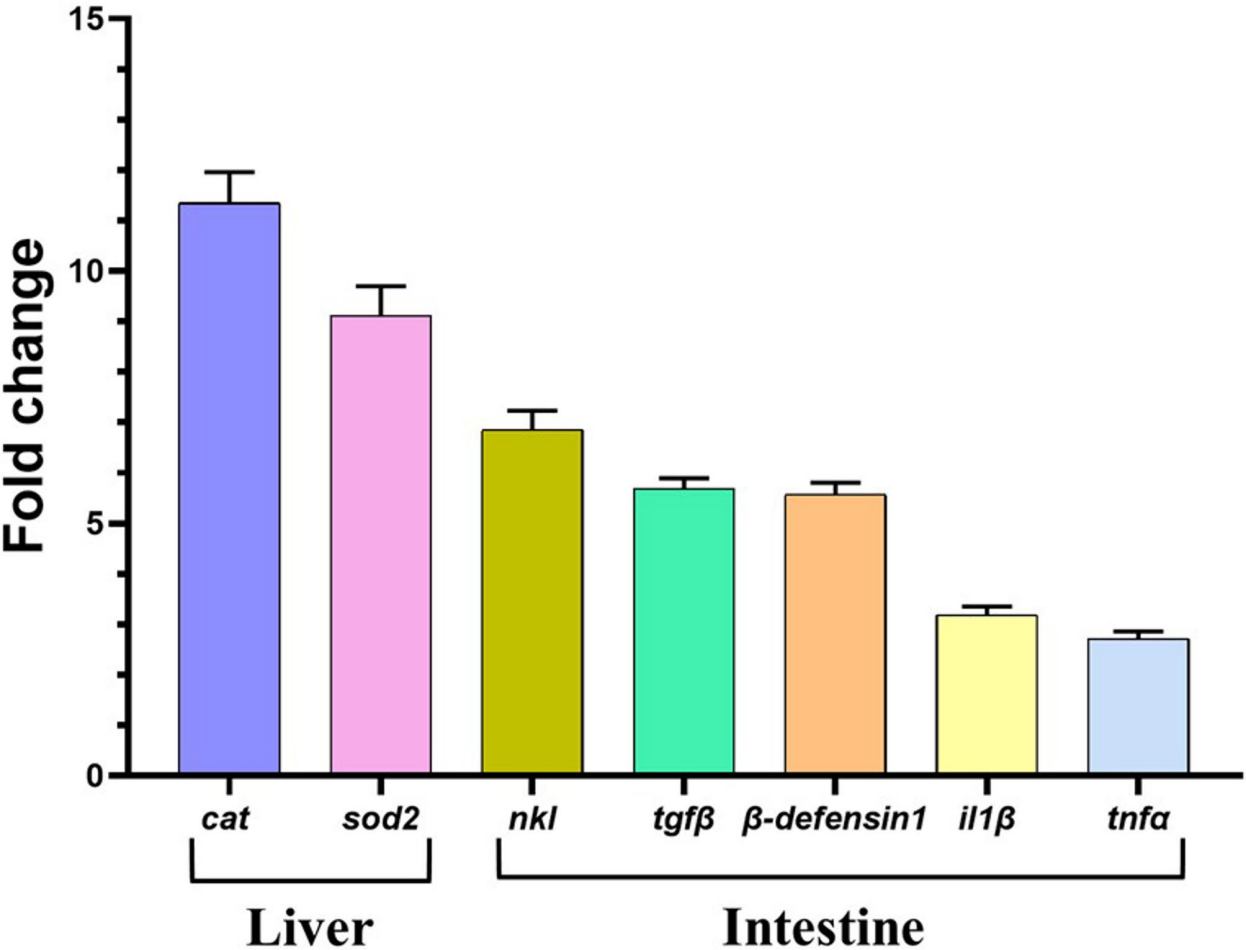
Maximum fold change of *il1β*, *tnfα*, *tgfβ*, *nkl*, *β-defensin 1*, *cat*, and *sod2* in the intestine and liver of Nile tilapia fed DPS-NPs (120 mg/kg) for one month. Values are expressed as mean ± standard error.

Overall, gene expression levels increased, peaking at 120 mg/kg. Among tissues, the liver exhibited the strongest expression of *sod2* and *cat*, whereas the intestine showed the greatest expression of the immune-related genes (Fig. 14).

### Histological evaluation

#### Intestine

**Fig. 15:**
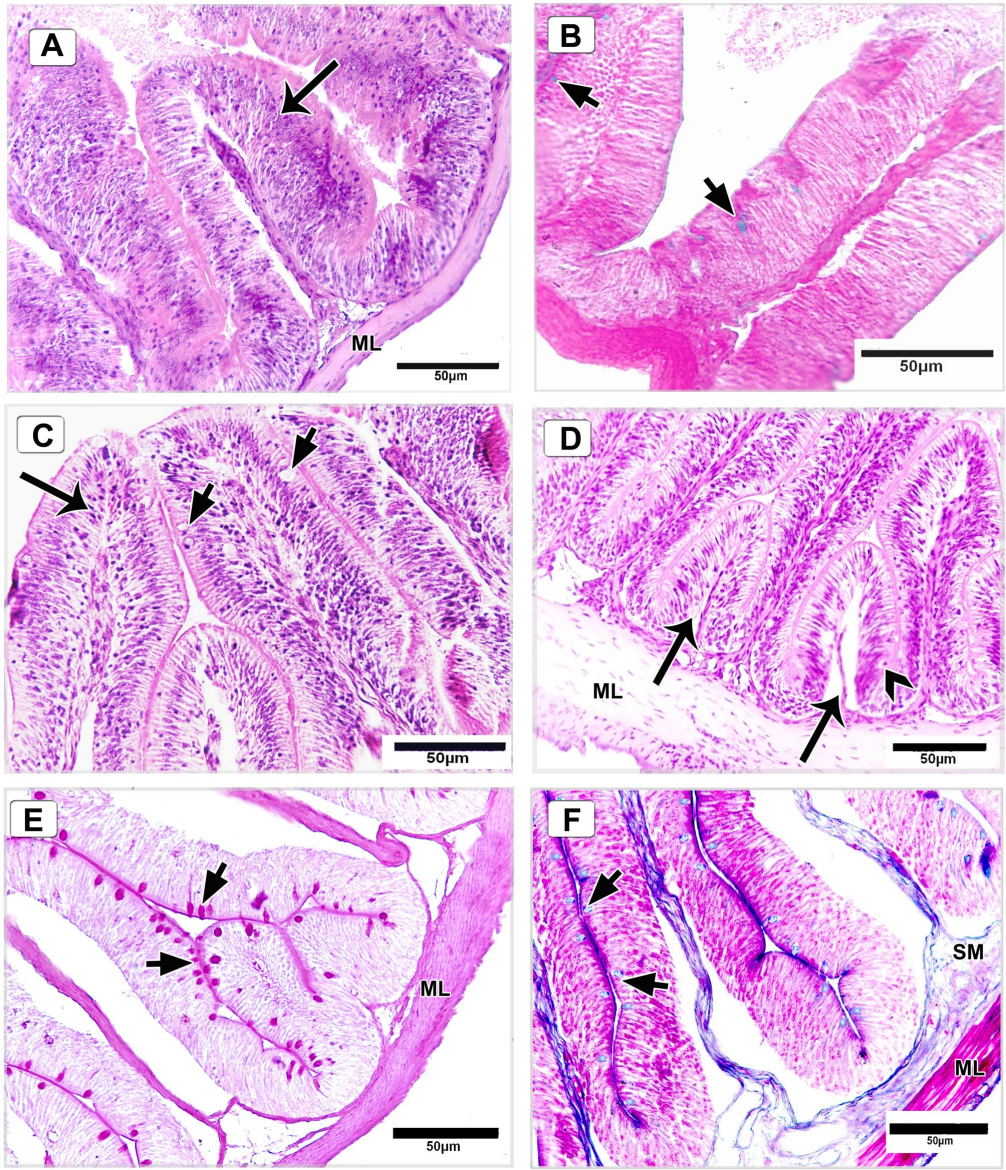
Histological sections of the intestine of Nile tilapia fed a basal diet (control) (**A**,** B**) and 120 mg/kg DPS-NPs (**C–F**). **A**,** B** Normal histoarchitecture with intact villi epithelium (arrow), sparse goblet cells (short arrows), and muscular layer (ML). **C** DPS-NPs supplemented fish show elongated, organized villi with brush border (arrow) and goblet cells (short arrows). **D** Villi bifurcation (arrows), mild intraepithelial lymphocytic infiltration (arrowhead), and intact ML. **E**,** F** Numerous goblet cells (short arrows) appear hyperactive, with strong periodic acid–Schiff (PAS; violet) and Masson’s trichrome (blue) staining. Submucosa (SM) shows increased vascularity. Sections: **A**,** C**,** D** H&E; **B**,** F** Masson’s trichrome; **E** PAS.

Histological examination of the intestine of Nile tilapia fed the basal diet (control group) revealed normal tissue architecture, with intact epithelial linings of the villi and a relatively low distribution of goblet cells (Fig. 15A, B). In contrast, fish supplemented with 120 mg/kg DPS-NPs displayed marked improvements in intestinal structure and cellular integrity. The mucosal layer exhibited elongated, well-organized villi with a clearly defined brush border (Fig. 15C). Evidence of villus bifurcation and mild intraepithelial lymphocytic infiltration was also observed (Fig. 15D). Goblet cells were abundantly distributed along the epithelial lining and appeared hyperactive, showing intense positive staining with both Periodic Acid-Schiff (PAS) (Fig. 15E) and Masson’s trichrome (Fig. 15F). The submucosa demonstrated increased vascularity (Fig. 15F), and the muscularis layer showed a notable increase in thickness compared with the control group (Fig. 15D–F).

Morphometric analysis confirmed a significant increase in intestinal villus height and muscular layer thickness in the DPS-NP–supplemented group. Villus height reached 634.28 ± 110.18 μm and muscular layer thickness measured 107.54 ± 30.46 μm, compared with 396.31 ± 49.14 μm and 30.14 ± 4.65 μm, respectively, in the control group.

#### Spleen

**Fig. 16:**
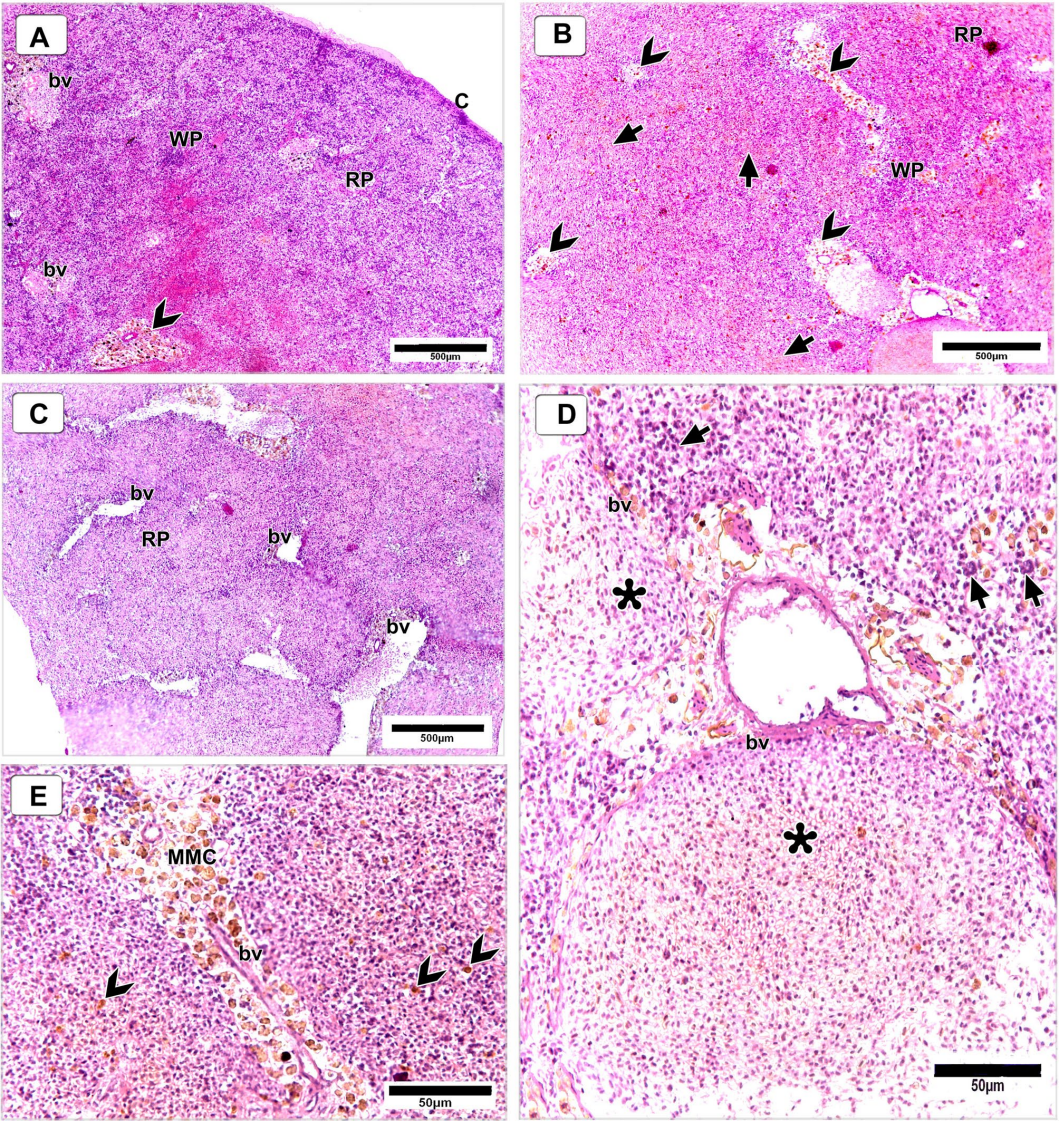
Histological sections of the spleen of Nile tilapia fed a basal diet (control) (**A**) and 120 mg/kg DPS-NPs (**B**–**E**). **A** Normal architecture with intact capsule **(B)**, moderately defined white pulp (WP), vascularized red pulp (RP) with orderly blood vessels (bv), and sparse melano-macrophage centers (arrowheads). **B**,** C** DPS-NPs group shows enlarged WP with lymphocyte aggregation, dilated RP, vascular proliferation, and abundant MMC clusters (arrowheads). **B** Hemosiderin deposits (short arrows) in parenchyma near MMCs. **D** WP densely populated with lymphocytes (short arrows); expanded bv packed with erythrocytes and hemosiderin (asterisks). **E** Extensive MMC proliferation near bv, indicating heightened phagocytic activity. Sections stained with H&E.

Microscopic examination of the spleen from Nile tilapia fed the basal diet (control group) revealed normal histoarchitecture. The splenic capsule was intact, and the white pulp was well organized, with moderately defined lymphoid tissue distributed throughout. The red pulp displayed typical vascularization with narrow, orderly blood vessels, while melano-macrophage centers (MMCs) were few in number and sparsely distributed (Fig. 16A).

In contrast, spleens from fish supplemented with 120 mg/kg DPS-NPs exhibited distinct histological changes indicative of immune stimulation. The white pulp was more prominently defined, with active lymphocyte aggregation, particularly around blood vessels. The red pulp showed marked vasodilation, vascular proliferation, and an increased number of blood vessels. Many enlarged vessels were densely packed with erythrocytes and hemosiderin deposits (Fig. 16B–D). The splenic parenchyma also showed pronounced hemosiderin accumulation, especially in areas associated with MMCs, suggesting enhanced immune activity and iron recycling.

Notably, the spleens of DPS-NP–supplemented fish displayed a proliferation of large MMC clusters, with numerous melano-macrophage cells dispersed throughout the tissue. These centers appeared more active, frequently aggregated near blood vessels, and reflected heightened phagocytic and immunological activity (Fig. 16E).

#### Liver

**Fig. 17:**
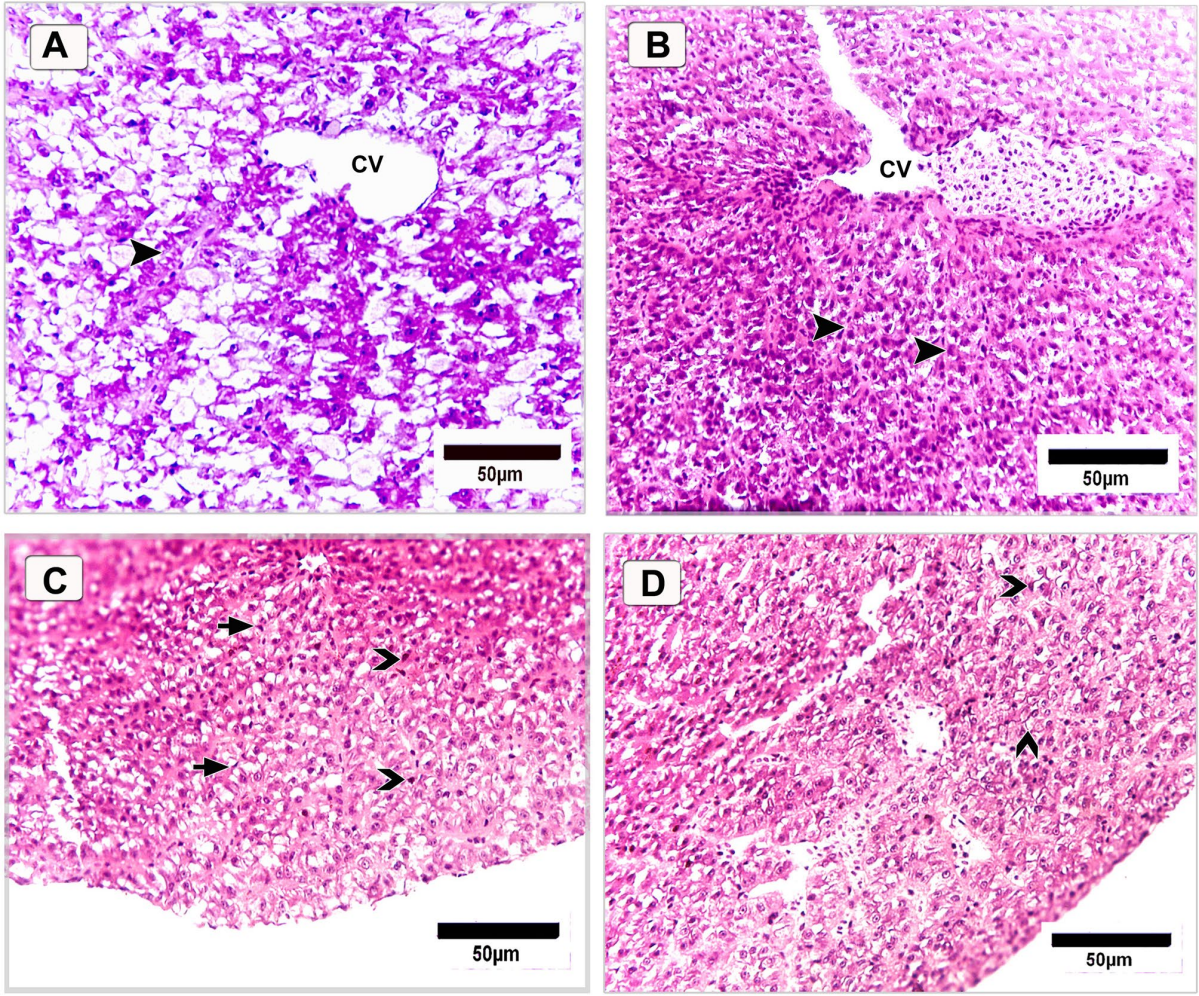
Histological sections of the liver of Nile tilapia fed a basal diet (control) (**A**) and 120 mg/kg DPS-NPs (**B–D**). **A** Control fish show hepatic cords (arrowhead) with cytoplasmic vacuolization. **B** DPS-NPs group shows restored hepatic structure with organized cords (arrowheads) around central vein (CV) and reduced vacuolization. **C** Increased Kupffer cells (short arrows) and melanomacrophages (arrowheads) with melanin deposits. **D** Numerous telocytes with elongated telopodes near blood vessels, suggesting a role in tissue repair and intercellular signaling. Sections stained with H&E.

In Nile tilapia fed the basal diet without supplementation (control group), liver histology revealed organized hepatic cords, with hepatocytes displaying some degree of cytoplasmic vacuolization (Fig. 17A). In contrast, fish supplemented with 120 mg/kg DPS-NPs exhibited partial restoration of normal hepatic structure. Hepatic cords were well-organized, radiating symmetrically around the central vein, and hepatocytes appeared metabolically active with markedly reduced vacuolization (Fig. 17B). A notable increase in active Kupffer cells was observed, indicating enhanced phagocytic activity. Furthermore, melanomacrophages were prominently distributed throughout the hepatic parenchyma and were characterized by conspicuous accumulations of melanin pigment (Fig. 17C). Numerous telocytes were also observed in close proximity to blood vessels, identifiable by their elongated telopodes extending through the hepatic tissue toward vascular structures (Fig. 17D), suggesting a potential role in intercellular communication and tissue remodeling.

## Discussion

Date palm seeds (DPS) are recognized as a rich source of essential nutrients and bioactive phytochemicals and are considered an affordable natural reservoir of antioxidants^25^. Previous studies have reported their anti-inflammatory properties both in vitro and in vivo^58^ as well as their capacity to enhance immune responses^59–61^. However, to date, no studies have examined the anti-inflammatory or immunostimulatory effects of DPS nanoparticles (DPS-NPs) in Nile tilapia, highlighting a gap in current research.

Serum biochemical and innate immune markers are widely used to assess the effects of feed additives in fish^62,63^. Serum proteins—primarily albumin and globulin—are major components of immune responses^64^. In the present study, dietary supplementation with DPS-NPs, particularly at 80 and 120 mg/kg, significantly increased serum total protein, albumin, and globulin compared with the control. Such increases are typically associated with stronger innate immune responses in fish^62^. Because serum proteins include both hepatic products and proteins involved in immune function, concentrations of total protein, albumin, and globulin are commonly interpreted as indicators of nutritional status, immune activity, and overall health. However, these parameters alone do not provide direct evidence of altered liver function or changes in protein synthesis rates^65–67^. Plasma albumin is synthesized primarily by the liver, whereas several globulin fractions originate from both hepatic and immune cells; thus, elevated globulin levels may reflect enhanced immune activity^68–70^. Consequently, while increases in serum protein fractions may be consistent with enhanced hepatic protein synthesis, they cannot be considered definitive evidence of this process. Nevertheless, the observed changes in serum protein levels are in agreement with previous studies in fish supplemented with phytogenic nanoparticles, which have reported improvements in biochemical and immune parameters^71–73^.

In this study, dietary supplementation with 120 mg/kg DPS-NPs significantly decreased serum AST, ALT, uric acid, and creatinine levels compared with the control group. Because AST and ALT are well-established indicators of hepatic integrity, their reduction reflects improved liver function and diminished hepatocellular injury^74^. Similarly, the observed decreases in uric acid and creatinine—key biomarkers of renal function—indicate a protective effect on kidney health^75,76^. Notably, these physiological improvements were accompanied by a significant reduction in serum MDA, a widely recognized marker of lipid peroxidation and oxidative stress. Lower MDA levels suggest that DPS-NPs effectively attenuated oxidative damage, thereby preserving both hepatic and renal integrity^76,77^. By limiting oxidative stress, DPS-NPs likely reduce the leakage of AST and ALT from hepatocytes and support renal filtration efficiency, preventing the accumulation of nitrogenous waste products.

The coordinated improvement in hepatic, renal, and oxidative stress biomarkers can be attributed to the high phenolic and flavonoid content of DPS, whose bioactivity is enhanced in nanoparticle form. These compounds function as potent free-radical scavengers and regulators of endogenous antioxidant defense systems, conferring pronounced hepato- and nephroprotective effects^5,78–80^. Moreover, nanoparticle formulation enhances the solubility, stability, and gastrointestinal absorption of bioactive phytochemicals. Owing to their nanoscale size, DPS-NPs readily traverse intestinal epithelial barriers via endocytosis and paracellular transport, resulting in increased intracellular availability of phenolic compounds^9,81,82^. This enhanced bioavailability enables DPS-derived antioxidants and immunomodulatory agents to more efficiently reach hepatocytes, renal tissues, and immune cells, thereby amplifying their systemic benefits. Collectively, the simultaneous reductions in AST, ALT, uric acid, creatinine, and MDA strongly support the use of dietary DPS-NPs as a functional feed additive for improving physiological performance in Nile tilapia.

Interleukin-1β (Il1β) and Tnfα are critical pro-inflammatory cytokines that mediate immune activation and regulation. They stimulate immune cells and enhance defense mechanisms such as phagocytosis, respiratory burst activity, and nitric oxide (NO) production^83,84^. Il1β is among the earliest cytokines expressed during immune responses, stimulating lymphocytes and promoting the release of additional cytokines^85^. Tnfα plays an essential role in cellular proliferation, differentiation, and cytokine stimulation^86^. TGFβ, by contrast, regulates immune homeostasis, balancing pro- and anti-inflammatory responses. It supports immune tolerance while orchestrating both initiation and resolution of inflammation by regulating chemotaxis, activation, and survival of diverse immune cells^87,88^.

In the current study, dietary supplementation with DPS-NPs induced the upregulation of key immune-related genes (*il1β*, *tnfα*, and *tgfβ*) across the examined tissues, with the exception of the 40 mg/kg dosage, indicating that DPS-NPs activate innate immune signaling pathways. The most pronounced responses were observed in the intestine at 120 mg/kg, where *il1β*, *tnfα*, and *tgfβ* were upregulated by 3.19-, 2.72-, and 5.7-fold, respectively, compared with the control group. Consistent immune-enhancing effects of date fruit extract have been reported in common carp (*Cyprinus carpio*), in which dietary supplementation significantly increased the expression of *il8*, *il10*, and *tgfβ* in the head kidney^89^.

In addition to immune stimulation, phenolic compounds present in DPS exert anti-inflammatory effects by modulating leukocyte migration and reducing inflammatory mediators such as lysozyme, nitric oxide, prostaglandin E₂, and MDA^90,91^. Notably, the upregulation of *tgfβ* suggests a regulatory function, contributing to the resolution of inflammation, tissue repair, and the maintenance of immune homeostasis. Comparable immunomodulatory effects have also been documented for other nanoparticle-based dietary supplements in fish, further supporting the role of DPS-NPs as effective immune modulators in aquaculture species^92^.

The intestine, as a primary site of antigen exposure, plays a central role in mucosal immunity and represents one of the first lines of defense against pathogen invasion^93^. Accordingly, the observed upregulation of cytokine genes in intestinal tissues following DPS-NPs supplementation indicates an enhanced mucosal immune response. Moreover, the spleen—a central lymphoid organ—and the liver, both widely recognized as indicators of systemic immune activation in teleost fish^94^, also exhibited significant cytokine upregulation at higher DPS-NPs doses. Collectively, these findings confirm the systemic immunostimulatory effects of DPS-NPs and suggest their potential as a functional feed additive to strengthen immune competence in Nile tilapia.

Antimicrobial peptides (AMPs) are key components of the innate immune system and act as a first line of defense against bacterial pathogens^95^. In fish, AMP expression can be triggered not only by pathogen exposure^47,96,97^, but also by dietary immunostimulants^95,98,99^. Monitoring AMP gene expression therefore provides valuable insights into the immunostimulatory potential of dietary supplements and their contribution to host defense.

In this study, dietary supplementation with DPS-NPs at 80 and 120 mg/kg significantly upregulated the expression of AMP genes (*β-defensin 1* and *nkl*) across the intestine, spleen, and liver. This upregulation may reflect shifts in leukocyte populations or changes in their activation status within these organs. Both β-defensins and Nkl are known not only for their direct antimicrobial activity—such as disrupting microbial membranes—but also for regulating immune cell recruitment and cytokine secretion. In addition, flavonoids present in DPS may further amplify these effects through direct antimicrobial activity and by activating immune cells, including natural killer cells, macrophages, and dendritic cells, particularly in immune-rich sites such as Peyer’s patches and the spleen^100^.

Key endogenous antioxidant enzymes such as superoxide dismutase (Sod) and catalase (Cat) play essential roles in cellular protection by converting reactive oxygen species (ROS) into less harmful forms^101–103^. In the present study, dietary supplementation with DPS-NPs significantly upregulated *sod2* and *cat* gene expression in Nile tilapia, while lowering MDA, a biomarker of oxidative stress. These results indicate that DPS-NPs enhance the antioxidant defense system, likely by activating enzymatic pathways that mitigate oxidative damage. Phytochemicals in DPS-NPs may play a direct role in this process by stimulating antioxidant-related gene expression.

Date palm seeds are widely recognized for their free radical–scavenging capacity and their ability to enhance cellular defense mechanisms against oxidative stress^104–107^, while nanoparticle-based delivery systems may further enhance these effects by facilitating more effective interactions with mitochondrial and cytosolic antioxidant pathways^108^. These properties are largely attributed to their rich content of bioactive constituents, including phenolic compounds, flavonoids, essential minerals, vitamins C and E, and dietary fiber^109,110^. Among these, flavonoids play a dual role by directly neutralizing reactive species and by modulating key enzymes involved in arachidonic acid metabolism, such as phospholipase A₂, cyclooxygenase, lipoxygenase, and nitric oxide synthase^111^. In support of these mechanisms, a recent study demonstrated that polyphenol-encapsulated date palm seed extracts promoted nuclear factor erythroid 2–related factor 2 (Nrf2) translocation and activated both Nrf2 and NF-κB signaling pathways in RAW264.7 macrophages, highlighting the capacity of date palm seed–derived compounds to elicit cytoprotective responses through Nrf2-dependent signaling^112^. Collectively, these findings indicate that date palm seed supplementation stimulates endogenous defense systems, as Nrf2 and NF-κB are central regulators of redox homeostasis and orchestrate multiple molecular pathways involved in limiting reactive oxygen species and maintaining physiological balance^113,114^.

Evidence from other species aligns with these findings. In common carp, DPS supplementation consistently elevated Sod and Cat activities while reducing lipid peroxidation, indicating enhanced oxidative defense^115^. In gilthead seabream (*Sparus aurata*), DPS supplementation, either alone or combined with probiotics, significantly increased the activities of Sod, Cat, and glutathione reductase (GR) in mucosal tissues such as skin and gills^116^. In alloxan-induced diabetic rats, supplementation with DPS powder improved tissue histology, reduced lipid peroxidation, and restored SOD, Cat, and GPx activities in the liver, kidneys, and pancreas^79^. Similarly, rats treated with a steeped DPS formulation showed improvements in SOD and GPx activity, comparable to vitamin C therapy^117^. Moreover, oral administration of aqueous DPS significantly reduced oxidative stress markers and increased SOD and Cat activities in the kidneys of carbon tetrachloride-exposed mice^75^.

The hierarchical clustering and correlation analyses provided valuable insights into the co-expression patterns of antioxidant- and immune-related genes. The heatmap with dendrogram revealed two distinct clusters, reflecting functional grouping based on expression similarity^118^. One cluster comprised the antioxidant markers *sod2* and *cat*, while the immune-related genes formed a separate cluster, further subdivided into two groups. *il1β* and *tnfα*, both pro-inflammatory cytokines, clustered together, suggesting potential co-regulation. The second subgroup (*tgfβ*, *nkl*, and *β-defensin 1*) comprised genes associated with immune modulation and antimicrobial defense. Their co-expression indicates complementary roles in maintaining mucosal immunity and tissue homeostasis. Overall, this separation highlights the divergent biological functions of these genes, with antioxidant enzymes mitigating oxidative stress and cytokines mediating immune and inflammatory responses.

The results also revealed that the liver exhibited the highest expression of antioxidant-related genes (*sod2* and *cat*), whereas the intestine showed the greatest upregulation of immune-related genes (*il1β*, *tnfα*, *tgfβ*, *β-defensin 1*, and *nkl*) compared with the spleen and liver. This pattern likely reflects organ-specific functions: the liver’s central role in metabolic detoxification and ROS scavenging, as it is the main site for antioxidant enzyme synthesis^101^, and the intestine’s role as the first site of interaction with dietary components, including nanoparticles. The intestinal mucosa is rich in immune cells and lymphoid tissues, enabling rapid production of pro-inflammatory cytokines (Il1β and Tnfα), regulatory mediators (Tgfβ), and AMPs (β-defensin 1 and Nkl), all of which are critical for mucosal immunity^119–121^. Thus, the enhanced intestinal expression of immune-related genes suggests that DPS-NPs modulate not only systemic antioxidant defense via hepatic responses but also gut-associated immune mechanisms essential for barrier integrity and pathogen defense.

Histological examination further revealed significant alterations in both the intestine and spleen, consistent with enhanced digestive and immune functions. In the intestine, DPS-NPs supplementation produced well-developed mucosal architecture, characterized by elongated villi and a marked increase in goblet cell density. Goblet cell hyperactivity suggests elevated mucous secretion and stimulation of mucosal immunity^122^, a critical response for preserving intestinal barrier integrity and resisting pathogen invasion. Enhancements in villus structure and goblet cell function^123^ therefore indicate that DPS-NPs may promote both digestive efficiency and local immune protection.

In the spleen, a pronounced increase in the number and clustering of melanomacrophage centers (MMCs), along with signs of their activation, reflected stimulated immune function. MMCs are established markers of immunological status in fish, contributing to phagocytosis, antigen processing, and iron recycling^124–126^. Their proliferation and activation in DPS-NPs-supplemented fish suggest upregulated immune activity that may enhance disease resistance and overall health. In addition, pronounced vascular proliferation in the red pulp likely indicates increased hematopoietic and circulatory activity, thereby supporting immune cell mobilization and efficient transport of nutrients and signaling molecules—further reinforcing the spleen’s role in systemic immunity^127^.

Enhancements in intestinal muscular layer thickness and villus height in DPS-NPs-treated tilapia also suggest improved absorptive capacity and barrier integrity. These morphological improvements are likely mediated by antioxidant and bioactive compounds in DPS-NPs, which may regulate cellular turnover, reduce oxidative stress, and promote mucosal regeneration. Together, the histological changes in spleen and intestine underscore the immunostimulatory potential of DPS-NPs. The vascular proliferation in the spleen, combined with the mucosal and structural improvements in the gut, reflect a coordinated upregulation of systemic and mucosal immune responses, supporting the hypothesis that DPS-NPs not only fortify innate immunity but also enhance overall physiological resilience.

In the liver, the proliferation of melanomacrophages and Kupffer cells further indicated heightened immune activity, likely driven by the immunomodulatory effects of DPS-NPs. These resident hepatic immune cells are crucial for pathogen clearance, antigen presentation, and inflammatory regulation. This observation is consistent with findings by Bakry et al.^99^ who emphasized their immunological importance in fish. Additionally, the presence of telocytes within the hepatic parenchyma supports improved liver tissue integrity and intercellular communication. Telocytes, interstitial cells with long cytoplasmic extensions, facilitate cell-to-cell signaling and structural support^128^. Their detection suggests a role in coordinating interactions among endothelial, immune, and hepatic cells, consistent with evidence that they form networks with immune cells^129^ and contribute to immune regulation^130^.

Importantly, although pro-inflammatory cytokines (*tnfα* and *il1β*) were upregulated, this immune activation did not translate into pathological inflammation. Histological examination of the intestine, liver, and spleen revealed preserved tissue architecture, indicating that DPS-NPs enhance immune responsiveness while maintaining tissue integrity and homeostasis. Mechanistically, this balanced response is likely mediated by their cytoprotective and immunomodulatory actions, as evidenced by reduced MDA levels, upregulation of the anti-inflammatory cytokine *tgfβ*, and improvements in hepatic and renal function. These findings are consistent with previous reports demonstrating that date palm seed–derived compounds, particularly phenolics and flavonoids, promote immune function through regulated cytokine modulation without inducing tissue damage^109,131,132^.

In fish, as in other vertebrates, a balanced immune response is essential for maintaining health. Although enhanced immune activity is required for effective pathogen defense, excessive or dysregulated activation can lead to pathological consequences^133,134^. In the present study, the observed increases in immune responses were modest and remained within physiological limits, indicating immune stimulation without adverse effects on health or normal physiological function.

Overall, the results demonstrate that DPS-NPs, particularly at dietary levels of 80 and 120 mg/kg, act as potent immunostimulants in Nile tilapia. Their efficacy appears to be mediated through modulation of immune-related genes and enhancement of both mucosal and systemic immunity, driven by polyphenols, polysaccharides, and flavonoid-regulated cytokine signaling pathways.

## Conclusions

This study demonstrates that dietary supplementation with DPS-NPs, particularly at 120 mg/kg, significantly enhances immune responses, mildly upregulates antioxidant gene expression, and improves organ function in Nile tilapia. The improvements observed in serum biochemical parameters, tissue histomorphology, and molecular markers underscore the potential of DPS-NPs as an effective functional feed additive in aquaculture. Collectively, these findings highlight the value of nanotechnology-based phytogenic supplements as a promising strategy to promote fish health, strengthen disease resistance, and support sustainable aquaculture practices.

## Data Availability

All data generated or analyzed during this study are included in this article.
